# Analysis of mean-field models arising from self-attention dynamics in transformer architectures with layer normalization

**DOI:** 10.1098/rsta.2024.0233

**Published:** 2025-06-05

**Authors:** Martin Burger, Samira Kabri, Yury Korolev, Tim Roith, Lukas Weigand

**Affiliations:** ^1^ Helmholtz Imaging, Deutsches Elektronen-Synchrotron, Hamburg, Germany; ^2^ Department of Mathematics, University of Hamburg, Hamburg, Germany; ^3^ Department of Mathematical Sciences, University of Bath, Bath, UK

**Keywords:** transformer architectures, self-attention dynamics, gradient flows, interaction energies, stationary states

## Abstract

The aim of this article is to provide a mathematical analysis of transformer architectures using a self-attention mechanism with layer normalization. In particular, observed patterns in such architectures resembling either clusters or uniform distributions pose a number of challenging mathematical questions. We focus on a special case that admits a gradient flow formulation in the spaces of probability measures on the unit sphere under a special metric, which allows us to give at least partial answers in a rigorous way. The arising mathematical problems resemble those recently studied in aggregation equations but with additional challenges emerging from restricting the dynamics to the sphere and the particular form of the interaction energy. We provide a rigorous framework for studying the gradient flow, which also suggests a possible metric geometry to study the general case (i.e. one that is not described by a gradient flow). We further analyse the stationary points of the induced self-attention dynamics. The latter are related to stationary points of the interaction energy in the Wasserstein geometry, and we further discuss energy minimizers and maximizers in different parameter settings.

This article is part of the theme issue ‘Partial differential equations in data science’.

## 1. Introduction

Transformer architectures and the associated (self-)attention dynamics gained strong interest recently due to the success of artificial intelligence relying on them in several applications. Examples include large language models such as GPT-4 [1], multimodal large language models such as vision language transformers [2,3], text-to-image generation like Stable Diffusion [4] and protein folding with AlphaFold [5,6], which won the Nobel Prize in Chemistry in 2024.

The practical success of transformers and (self-)attention dynamics calls for developing detailed mathematical understanding which started recently in [7–19].

An interesting viewpoint on such dynamics is to interpret it as an interacting particle system [8,20,21], which allows for natural continuous-time and mean-field limits. The latter approach already provided valuable insights into feed-forward neural networks and their training dynamics (cf. [22,23]). In the context of transformers, this viewpoint also provides interesting (so far formal [9]) connections to gradient flows and the minimization of interaction energy for the particle measures. The latter is a topic of great recent interest due to various applications in biology and social interactions. Indeed, the self-attention dynamics in transformers share certain mathematical similarities with models used in opinion formation, which also exhibit similar emergence of clusters in certain cases [24–26]. In this work, we focus on cluster formation in the infinite time horizon. However, we note that the formation of metastable states is of special interest. For the case of isotropic interaction, metastability was studied in [27,28].

In this article, we proceed with the work in [9] on analysing transformer dynamics with layer normalization, focusing in particular on the case when the underlying dynamics has a gradient flow structure. Indeed, the continuum limit of the self-attention dynamics leads to a Wasserstein-type gradient flow for probability measures on the unit sphere S of the form


(1.1) $$\partial_{t}\mu_{t}=\nabla_{S}\cdot (\mu_{t}m_{\mu_{t}}\nabla_{S}E^{\prime }(\mu_{t})),$$

where $\nabla_{S}$ and $\nabla_{S}\cdot$ are the tangential gradient and divergence, respectively, and $m_{\mu }=\frac{1}{E^{\prime }(\mu )}$ is a non-local mobility. The underlying energy in this case is of the form


(1.2) $$E(\mu )=\int_{S}\int_{S}e^{x\cdot Dy}\;d\mu (x)\;d\mu (y),$$

with D being a symmetric matrix and $E^{\prime }$ denoting its first variation. Since D is symmetric and hence diagonalizable, we can equivalently assume that D is a diagonal matrix, since we can use an orthogonal diagonalization and a corresponding transfer of variables to the eigenvectors, which leaves the unit ball unchanged. This will be used in several instances to simplify notation. It also permits a more detailed study of stationary patterns, in particular minimizers and maximizers of the energy.

Compared to the existing literature on such gradient flows, there are three distinct features that motivate our study, namely:

— restriction of the dynamics to the unit sphere (a consequence of the layer normalization);
— non-local mobility (a consequence of the self-attention mechanism), which is related to but still distinctly different from other variations of Wasserstein gradient flows studied recently (cf. [29–32]);
— multiplicative coupling of states in the interaction energy, as opposed to commonly used interaction potentials depending only on the difference of the states (cf., e.g. [33–38]).

We make the gradient flow, formally introduced in [9], rigorous, showing that the transport distance with non-local mobilities is well defined, studying energy dissipation properties of the associated gradient flow and describing the large-time behaviour of the dynamics, specifically the convergence to stationary solutions, at least along subsequences. We further carry out a detailed study of energy minimizers and maximizers of E (extending the previously studied case of D being a multiple of the identity) as well as stationary points of the energy in a Wasserstein setting, which we prove to be equivalent to stationary solutions of the dynamics. For the energy minimizers, we obtain an interesting picture depending on the structure of D :

— If there is a positive eigenvalue that is the eigenvalue of maximal absolute value, then a Dirac delta concentrated in the direction of a corresponding eigenvalue is a maximizer.
— If the smallest eigenvalue is negative, then only a Dirac delta concentrated in the direction of a corresponding eigenvalue is a minimizer.
— If the smallest eigenvalue is zero, then any measure concentrated on the null space of D is a minimizer.
— Dirac deltas concentrated in directions of arbitrary eigenvectors are stationary points. We also find some convex combinations of Dirac deltas being stationary points.
— If the smallest eigenvalue is positive, we conjecture that the minimizer of the energy has full support on the unit sphere. To obtain some insight, we carry out a second-order asymptotic analysis of the minimizers for D being a small perturbation of the identity.

We support our theoretical findings with several computational experiments and investigate the cases when the energy minimizers or maximizers cannot be characterized explicitly.

The rest of this work is organized as follows. In the remainder of the introduction, we recapitulate the simplified softmax transformer model introduced in[8], with additional layer normalization as considered in [9]. In §2, we provide a rigorous derivation of the gradient flow induced by the considered model. Sections 3 and 4 are dedicated to characterizing optimizers or stationary points of the studied energy, respectively. We support our findings by numerical experiments in §5 and summarize our results in §6.

### (a) Self-attention

Transformer architectures [39] were developed in the field of natural language processing. Here, the input is usually a sentence, which is decomposed into a sequence of tokens (e.g. words or syllables). Each token (possibly along with its position in the sentence) is represented as a vector in a high-dimensional vector space. Apart from a conventional feed-forward component, the main feature of a transformer layer is the so-called attention mechanism. This mechanism implements interactions between tokens and was first introduced in [40] in the context of neural machine translation as an alternative to encoder–decoder approaches, the performance of which often deteriorates for large input lengths due to the use of latent representations of fixed dimensions.

Like [9], we shall focus on a simple yet widely used form of attention, the so-called self-attention. It can be formalized as follows: consider an input sequence $X=[X_{i}{]}_{i=1}^{N}\in {\mathbb{R}}^{N\times n}$ , where each $X_{i}\in {\mathbb{R}}^{n}$ represents an n -dimensional token and N denotes the number of tokens. The self-attention matrix $A\in {\mathbb{R}}^{N\times N}$ is given by


(1.3) $$A_{ij}=\frac{\exp\left(X_{i}\cdot DX_{j}\right)}{\sum_{k=1}^{N}\exp\left(X_{i}\cdot DX_{k}\right)},$$

where we assume $D\in {\mathbb{R}}^{n\times n}$ to be symmetric. The latter property does not necessarily hold for learned parameters in transformer architectures, but we expect the symmetric part to determine the asymptotic behaviour of the self-attention dynamics. Since the symmetry of D allows one to interpret the dynamics as a gradient flow corresponding to a certain interaction energy, as observed in [9], it will allow us to analyse the asymptotic behaviour for this subclass; the study of the general case is left for future research. An important example of non-symmetric interaction is given by masked attention, which can be used to model causality. We refer to [41–43] for a mean-field interpretation of such dynamics.

By definition, the matrix A is stochastic, i.e. each of its rows is a probability vector. Roughly speaking, the attention matrix determines how strongly a token is influenced by each other token. To determine *how* tokens influence each other, another matrix $V\in {\mathbb{R}}^{n\times n}$ , called the value matrix, is used. The influence of $X_{j}$ on $X_{i}$ can then be written as $A_{ij}\;VX_{j}$ and the self-attention layer $A:{\mathbb{R}}^{N\times n}\to {\mathbb{R}}^{N\times n}$ is given by


(1.4) $$A(X)={\left[X_{i}+\sum_{j=1}^{N}A_{ij}VX_{j}\right]}_{i=1}^{N}.$$

For our purposes, we assume V=D or V=−D since, in this case, one can show that the particles move along a gradient flow. The general case is the subject of future work.

### (b) Normalization method

The normalization of intermediate values is a common practice in machine learning models. In the context of neural networks, so-called batch normalization [44] is a popular method to prevent gradients from blowing up and thus to stabilize (and to improve) the training. Since this form of normalization uses information from the entire training batch, [45] proposes layer normalization (LayerNorm), which translates the mean of an intermediate vector to zero and divides it by its standard deviation, and therefore does not depend on any other vector in the batch. While the original implementation of the transformer [39] uses LayerNorm, some of the more recent publications (e.g. Llama, [46]) use a simplified version called Root Mean Square Layer Normalization (RMSNorm) proposed in [47]. Up to a multiplication with learned weights $[g_{i}{]}_{i=1}^{n}$ , called gain parameters, RMSNorm performs a projection on to the unit sphere $S^{n-1}$ (where in the following, we shall suppress the superscript and simply write S ). More precisely, for $x\in {\mathbb{R}}^{n}$ we write


$$\begin{array}{r} \mathrm{RMSNorm}(x{)}_{i}=g_{i}\frac{x_{i}}{\|x{\|}_{2}}, \end{array}$$

where, in practice, a division by zero is circumvented by adding a small value ϵ>0 into $\|x{\|}_{2}$ . In our setting, we can assume the norm to be strictly positive as we consider the dynamics in continuous time. Following the setting of [9], we focus on RMSNorm with fixed gain parameters $g_{i}=1$ for all i=1,…n and denote the projection on to the unit sphere for $x\in {\mathbb{R}}^{n}\setminus \left\{0\right\}$ by


$$\begin{array}{r} \Pi (x)=\frac{x}{\|x{\|}_{2}}. \end{array}$$

### (c) Simplified transformer layer and time-continuous dynamics

Combining the attention layer with a normalization layer, we arrive at the following update step:


$$\begin{array}{r} X\leftarrow \Pi (A(X)), \end{array}$$

where the projection is applied vector-wise to each row of A(X) . For the sake of our analysis, we shall deviate from typical practical implementations of transformers and consider the architecture to be a composition of such layers which all share the same matrices D and V in equations (1.3) and (1.4). In [9], it was proposed to study the continuum limit of these updates. This approach has become a popular tool for analyzing residual neural networks [48]: as discussed from various perspectives, e.g. in [49–52], the skip connections (i.e. the residual components) of the residual neural network architecture make it possible to interpret it as a forward Euler discretization of an ordinary differential equation. Introducing a time variable t>0 and a small time increment Δt>0 , we get


(1.5) $$X_{i}(t+\Delta t)=\Pi \left(X_{i}(t)+\Delta t\sum_{j=1}^{N}A_{ij}(t)VX_{j}(t)\right),\;i=1,\ldots ,N.$$

At this point, the residual component is hidden in the attention layer and cannot easily be extracted since the projection is nonlinear. In the continuous time limit Δt→0 , remembering that Π(x)=x for any x∈S , we arrive at the following system of differential equations:


(1.6)X˙i(t)=⟨∇xΠ(Xi(t)),∑j=1NAij(t)VXj(t)⟩,i=1,…,N,

where the spatial derivatives are understood as derivatives in ${\mathbb{R}}^{n}$ . With a simple computation, one can further show that for any x∈S and $z\in {\mathbb{R}}^{n}$ it holds that


$$\begin{array}{r} \langle \nabla_{x}\Pi (x),z\rangle =P_{x}^{⟂}(z), \end{array}$$

where, following [9], we define $P_{x}^{⟂}(z)=z-x\cdot z\;x$ . Substituting this into equation (1.6), we arrive at the following dynamics:


$$\left\{ \begin{array}{l} {\dot{X}}_{i}(t)=P_{X_{i}(t)}^{⊥}\left(\sum_{j=1}^{N}A_{ij}(t)VX_{j}(t)\right),\;\text{(1.7a)}\\ X_{i}(0)=X_{0,i}\in S,\;\text{(1.7b)} \end{array}\right.$$

which serve as a starting point of [9].

### (d) Interpretation as an evolution of measures

Instead of studying the dynamics of distinct particles, [9] propose to view equation (1.7) as an evolution of an empirical measure


$$\mu_{t}=\frac{1}{N}\sum_{i=1}^{N}\delta_{X_{i}(t)}.$$

The right-hand side of equation (1.7a) can be understood as an integral with respect to $\mu_{t}$ ; for a generic probability measure μ , this can be written as a measure-dependent velocity field:


(1.8) $$\begin{array}{lrr} & V[\mu ](x)=\frac{P_{x}^{⟂}\left(\int_{S}e^{x\cdot Dy}\;Vy\;\;d\mu (y)\right)}{\int_{S}e^{x\cdot Dy}\;\;d\mu (y)}, & \end{array}$$

and equation (1.7a) turns into $\dot{X_{i}}(t)=V[\mu_{t}](X_{i}(t))$ . With this notion, we recover the weak continuity equation formulated in [9]: for any test function $\varphi \in C^{1}(S\times [0,T])$ , one has


ddt∫Sϕ(t,x)dμt(x)=ddt1N∑i=1Nϕ(t,Xi(t))=1N∑i=1N∂tϕ(t,Xi(t))+⟨∇xϕ(t,Xi(t)),V[μt](Xi(t))⟩(1.9)=∫S∂tϕ(t,x)+⟨∇xϕ(t,x),V[μt](x)⟩dμt(x),

where, in this case, the spatial derivatives of φ have to be understood as derivatives on S .

Similarly, Geshkovski *et al*. [9] propose the interaction energy in equation (1.2), which for an empirical measure $\mu_{t}$ reduces to


$$E\left(\mu_{t}\right)=\sum_{i,j=1}^{N}e^{X_{i}(t)\cdot DX_{j}(t)}.$$

In this discrete case, a straightforward application of the chain rule and a reordering of the terms yields


$$\frac{d}{dt}E\left(\mu_{t}\right)=2\sum_{i=1}^{N}\left(\sum_{j=1}^{N}e^{X_{i}(t)\cdot DX_{j}(t)}DX_{j}(t)\right)\cdot {\dot{X}}_{i}(t).$$

Under our assumption that the value matrix is given by V=±D , we see that, up to an application of $P_{X_{i}(t)}^{⟂}$ and a division by $\sum_{j=1}^{N}e^{X_{i}(t)\cdot DX_{j}(t)}$ , the term in the brackets is given by $\dot{X_{i}}(t)$ . Since $P_{x}^{⟂}(z)\cdot z=P_{x}^{⟂}(z)\cdot P_{x}^{⟂}(z)$ for any x∈S , $z\in {\mathbb{R}}^{n}$ , we have that


$$\frac{d}{dt}E\left(\mu_{t}\right)=\pm 2\sum_{i=1}^{N}{\left\|{\dot{X}}_{i}(t)\right\|}^{2}\sum_{j=1}^{N}e^{X_{i}(t)\cdot DX_{j}(t)}\;\underset{\leq }{\geq }0,$$

and hence the energy E increases ( V=D ) or decreases ( V=−D ) monotonously along the trajectory of $\mu_{t}$ . A formal derivation of the above formulae for general probability measures on smooth manifolds is provided in §2.

Let us mention that problems with similar energies as E have been studied in the past. The most prominent is an interaction energy with respect to D with a non-local interaction kernel depending on x−y . Choosing the kernel as Gaussian with covariance matrix $D^{-1}$ (which makes sense only if D is positive definite) results in


(1.10)Einter(μ)=∫S∫Se−12(x−y)⋅(D(x−y))dμ(x)dμ(y).

For D=±Id⁡ , the minimizers and maximizers of the expressions in equations (1.2) and (1.10) are equivalent as $\mp \frac{1}{2}(x-y)\cdot (x-y)=\mp \frac{1}{2}(x\cdot x+y\cdot y)\pm x\cdot y=\mp 1+\pm x\cdot y$ for all x,y∈S . The important difference between equations (1.2) and (1.10) is the rotation-(in)variance of the interaction functions $e^{x\cdot (Dy)}$ and $e^{-\frac{1}{2}(x-y)\cdot (D(x-y))}$ . In the general case, this is not true, but we shall use an analogy to the interaction energy to rewrite


$$E(\mu )=e^{\lambda }\int_{S}\int_{S}e^{-\frac{\lambda }{2}|x{|}^{2}-\frac{\lambda }{2}|y{|}^{2}+x\cdot Dy}\;d\mu (x)\;d\mu (y)=e^{\lambda }\int_{S}\int_{S}e^{-\frac{\lambda }{2}|x-y{|}^{2}+x\cdot ((D-\lambda \mathrm{Id})y)}\;d\mu (x)\;d\mu (y).$$

### (e) Understanding x⋅Dy on the sphere

For our further analysis, it is crucial to understand the implications of restricting the problem to the unit sphere and the behaviour of the bilinear form x⋅Dy on it. For D=Id⁡ , it is clear that the minimizer of $f_{y}(x)=x\cdot Dy$ is given by x=−y and the maximizer by x=y . This changes for a general D and as a result, the minimizer of the energy in equation (1.2) is not given by the uniform distribution on S anymore. For a diagonal matrix D , the maximizer/minimizer of $f_{y}$ for a fixed y∈S with Dy≠0 is given by $x_{\pm }=\pm \frac{Dy}{\left\|Dy\right\|}$ . Therefore, we know that x⋅Dy=0 if and only if $x\cdot x_{\pm }=0$ (same for > and < ). For Dy=0 , we already have $f_{y}(x)=0$ for any x∈S , i.e. each point is a minimizer, maximizer and orthogonal to y w.r.t. D . A further consequence is that


$$\begin{array}{r} \max_{x,y\in S}\;x\cdot Dy=\max_{y\in S}\;\frac{Dy\cdot Dy}{\left\|Dy\right\|}=\max_{y\in S}\|Dy\|=|\lambda |, \end{array}$$

where λ denotes the eigenvalue of maximum absolute value of D . We further note that all of the following results on minimizers/maximizers as well as stationary points of $E_{D}$ can be generalized to probability measures concentrated on an ellipsoid instead of a sphere. To see this, we consider the ellipsoid


$$\begin{array}{r} CS=\left\{x\in {\mathbb{R}}^{n}:\;\|C^{-1}x\|=1\right\}, \end{array}$$

where $C\in {\mathbb{R}}^{n\times n}$ is invertible, and the corresponding energy


$$\begin{array}{r} E_{D}^{C}(\mu )=\int_{CS}\int_{CS}e^{x\cdot Dy}\;\;d\mu (x)\;\;d\mu (y). \end{array}$$

Since C is invertible, any measure μ is uniquely determined by the pushforward measure $\nu =C_{\#}^{-1}\mu$ , as $\mu =C_{\#}\nu$ . Thus, we can rewrite the energy as


$$\begin{array}{r} E_{D}^{C}(\mu )=\int_{S}\int_{S}e^{Cx\cdot DCy}\;\;d\nu (x)\;\;d\nu (y)=E_{C^{T}DC}(\nu ), \end{array}$$

and equivalently optimize the energy $E_{C^{T}DC}$ on the sphere. A special case that leads to measures concentrated on an ellipsoid corresponds to RMSNorm normalization with non-vanishing gain parameters $g_{i}\neq 0$ . In this case, the ellipsoid is given by GS , where G is a diagonal matrix with entries $[g_{i}{]}_{i=1}^{n}$ .

## 2. Gradient flow

As shown above, the particle dynamics can be ‘lifted’ by the use of empirical measures to the space of probability measures P(S) over the sphere. As mentioned in [9, Remark 3.3], for arbitrary probability measures, the connection between the partial dynamics and a corresponding continuity equation can be made by a mean field limit approach. Hence, instead of the particle dynamics, one can study the continuity equation:


(2.1) $$\begin{array}{rl} \partial_{t}\mu +div(V[\mu ]\mu )=0 & \;\text{on }[0,T]\times S,\\ \mu_{|t=0}=\mu (0) & \;\text{on }S, \end{array}$$

with the velocity field given by equation (1.8), which holds in the sense of distributions. Note that, in this section, we scale the energy by a factor of 1/2 to be consistent with [9]. It was remarked in [9, ch. 3.3] that for V=±D , the energy,


$$E(\mu )=\pm \frac{1}{2}\int_{S}\int_{S}e^{x\cdot Dy}\;\;d\mu (x)\;\;d\mu (y),$$

is monotonic along these dynamics, and the partial differential equation (2.1) can be interpreted as a gradient flow for a modified optimal transport distance. However, as the authors of [9] acknowledge, there is a gap in the literature that prevents them from making this observation rigorous.

In this section, we aim to close this gap. We show that P(S) equipped with this new distance is a geodesic space with properties similar to the classical 2 -Wasserstein space and prove that solutions of equation (2.1) are curves of maximal slope of E with respect to this distance and thus satisfy the energy dissipation equality


$$\begin{array}{r} \frac{\;d}{\;dt}E(\mu_{t})=-\int_{S}\int_{S}e^{x\cdot Dy}\;d\mu_{t}(y)\;|V[\mu_{t}](x){|}^{2}\;d\mu_{t}(x)\;\text{for a.e. }t. \end{array}$$

Finally, we study the long-time behaviour of the dynamics and show that subsequences of the flow converge to stationary points of the energy E .

Let us mention that the basic analysis of this section related to the novel transport distance can be generalized in a rather straightforward way to the more general case of D being non-symmetric and can thus provide the basis for future analysis of the non-gradient flow case with V arbitrary and D non-symmetric.

### (a) Continuity equation on manifolds

Let M be a compact n -dimensional Riemannian manifold without a boundary, e.g. the sphere $S\subset {\mathbb{R}}^{n}$ . The tangent bundle $TM={⊔}_{x\in M}T_{x}M$ is given by the disjoint union of all tangent spaces of all x∈M . We denote by P(M) the space of Borel probability measures on M , equipped with the standard narrow topology (e.g. [53, ch. 5.1]). The symbol ⇀ is used to indicate convergence in this topology. Let I=(0,T) be an open interval, $\mu :t\to \mu_{t}\in P(M)$ a narrowly continuous curve and $V:(x,t)\in M\times I\mapsto v_{t}(x)\in TM$ a Borel velocity field such that $\int_{0}^{T}\int_{M}|v_{t}(x)|\;d\mu_{t}\;dt<\infty$ . The continuity equation holds in the sense of distributions if


(2.2) $$\begin{array}{lrr} & \int_{\left(0,T\right)}\int_{M}\partial_{t}\varphi (x,t)+\langle D\varphi (x,t),v_{t}(x)\rangle \;d\mu_{t}\;dt=0,\;\forall \varphi \in C_{c}^{1}(M\times (0,T)). & \end{array}$$

Here, D denotes the differential on the manifold M . Sometimes, we shall use $D_{x}$ to clarify with respect to which variable the differential is taken. We define the set of solutions to the continuity equation as follows:


$$\begin{array}{r} CE(0,T):=\left\{(\mu ,v):\;\begin{array}{c} \mu :I\mapsto P(M)\text{ is narrowly continuous},\\ \int_{0}^{T}\int_{M}|v_{t}(x)|\;d\mu_{t}\;dt<\infty ,\\ (\mu ,v)\text{ satisfy the continuity equation,} \end{array}\right\} \end{array}$$

Furthermore, we define CE(0,T;ν→η) as the subset (μ,v) such that $\mu_{0}=\nu$ , $\mu_{T}=\eta$ . For more details, we refer to appendix A(a).

### (b) Distance

To interpret equation (2.1) as a gradient flow on P(M) , we need to modify the well-known dynamic formulation of the 2 -Wasserstein distance [54] and introduce the following mobility:


$$\begin{array}{r} m_{\mu }(x)=\int_{M}K(x,y)\;d\mu (y). \end{array}$$

With this, the modified transport distance between $\mu_{0},\mu_{1}\in P(M)$ is defined as follows (see [9, Section 3.4.2]):


(2.3) $$\begin{array}{lrr} & W_{m,2}^{2}(\mu_{0},\mu_{1})=\inf\left\{\int_{0}^{1}\int_{M}m_{\mu_{t}}(x)|v_{t}(x){|}^{2}\;d\mu_{t}(x)\;dt:(\mu ,v)\in CE(0,1;\mu_{0}\to \mu_{1})\right\}. & \end{array}$$

For K≡1 , we recover the classical 2 -Wasserstein distance. The dynamic (2.1) corresponds to the kernel $K(x,y)=e^{x\cdot Dy}$ *,* but for the sake of generality, we carry out the analysis for a more general class of kernels K .

**Assumption 1.** The kernel K(x,y)∈C(M×M) is continuous, and there exists a constant C>0 such that K(x,y)⩾C for all x,y∈M .

**Remark 2.1.** *The assumption that* K *is bounded from below is vital for our analysis and covers the cases of interest in this article. Nonetheless, it would be interesting to see whether this assumption can be relaxed. For example, instead of a compact manifold* M *, we could consider* ${\mathbb{R}}^{d}$ *as the underlying space and take* K *to be a Gaussian or a bounded confidence kernel* $K(x,y)=1_{\left|x-y\right|⩽1}$ *as studied in [* 55 *]*.

As the next theorem shows, the infimum in equation (2.3) is actually attained by some $(\mu ,v)\in CE(0,1;\mu_{0}\to \mu_{1})$ . The proof can be found in appendix A(b).

**Theorem 2.2** (Existence of minimizers). *For every pair* $\mu_{0},\mu_{1}\in P(M)$ *with* $W_{m,2}(\mu_{0},\mu_{1})<+\infty$ , *there exists a couple* (μ,v)∈CE(0,1) *such that*


$$\begin{array}{r} W_{m,2}^{2}(\mu_{0},\mu_{1})=\int_{0}^{1}\int_{M}m_{\mu_{t}}(x)|v_{t}(x){|}^{2}\;d\mu_{t}(x)\;dt. \end{array}$$

*Furthermore, such minimizers can be equivalently characterized as those of*


(2.4)Wm,2(μ0,μt)=inf{∫0T(∫Mmμt(x)|vt(x)|2 dμt(x))12 dt:(μ,v)∈CE(0,T;μ0→μT)}.

Using the theorem above, it is easy to show that $W_{m,2}$ is a distance on P(M) .

**Theorem 2.3.** *The space* P(M) *equipped with* $W_{m,2}$ *is a complete metric space and its topology is equivalent to the one induced by the* 2 *-Wasserstein distance which, since* M *is compact, is equivalent to the topology of narrow convergence*.

*Proof.* First, we check that $W_{m,2}$ is a distance. Indeed, (i) symmetry follows from simply rescaling time by $\tilde{t}:t\in [0,T]\;\mapsto \;T-t\in [0,T]$ ; (ii) definiteness: Since $m_{\mu_{t}}$ is bounded from below, $W_{m,2}(\mu ,\nu )=0$ implies that $v_{t}=0$ for μ -a.e. (p,t)∈M×(0,T) . Thus by equation (A 3) μ=ν ; (iii) the triangle inequality follows from the characterization in equation (2.4) and the gluing property from proposition A.1. To show the equivalence of the distances, we observe that by assumption 1, K(x,y)⩾C and since M×M is compact and K(x,y) is continuous, we can also find a $\tilde{C}$ such that $K(x,y)⩽\tilde{C}$ . This implies that


$$\begin{array}{r} \frac{1}{C}W_{2}(\mu ,\nu )⩽W_{m,2}(\mu ,\nu )⩽\tilde{C}W_{2}(\mu ,\nu )<+\infty \;\forall \mu ,\nu \in P(M), \end{array}$$

and the distances are equivalent. Since $\left(P(M),W_{2}\right)$ is complete, $\left(P(M),W_{m,2}\right)$ has to be complete as well.∎

Let us recall that in a general complete metric space (X,d) , a curve γ:[0,T]→X is called absolutely continuous if there exists a function $m\in L^{1}(0,T)$ such that


(2.5) $$\begin{array}{r} d(\gamma_{s},\gamma_{r})⩽\int_{s}^{r}m(t)\;dt\;\forall s,r\in [0,T]\text{ with }\;s⩽r. \end{array}$$

For an absolutely continuous curve γ(t) , its metric derivative is defined by


$$\begin{array}{r} |\dot{\gamma }|(t):=\lim_{h\to 0}\frac{d(\gamma_{t+h},\gamma_{t})}{h}, \end{array}$$

and it exists for a.e. t∈(0,T) . It can be shown that $\left|\dot{\gamma }\right|$ is minimal in the sense that for all m(t) satisfying equation (2.5), it holds that $|\dot{\gamma }|(t)⩽m(t)$ for a.e. t∈(0,T) . The next lemma, which is proven in appendix A(c), characterizes absolutely continuous curves in $\left(P(M),W_{m,2}\right)$ .

**Lemma 2.4.** *Let* $\mu_{t}$ *be an absolutely continuous curve w.r.t*. $W_{2,m}$ . *Then there exists a Borel velocity field* $(v_{t}{)}_{t\in (0,T)}$ *such that* (μ,v)∈CE(0,T) *and*


$${\left(\int_{M}m_{\mu_{t}}(x)|v_{t}(x){|}^{2}\;d\mu_{t}(x)\right)}^{1/2}=|\dot{\mu }|(t)\;for\;a.e.\;t\in (0,T).$$

*Conversely, if* (μ,v)∈CE(0,T) *and* $\int_{0}^{T}{\left(\int_{M}m_{\mu_{t}}|v_{t}{|}^{2}\;d\mu_{t}\right)}^{1/2}\;dt<+\infty$ *then* $t\to \mu_{t}$ *is absolutely continuous and*


$$|\dot{\mu }|(t)⩽{\left(\int_{M}m_{\mu_{t}}(x)|v_{t}(x){|}^{2}\;d\mu_{t}(x)\right)}^{1/2}\;for\;a.e.\;t\in (0,T).$$

A metric space is called a length space if


$$d(x,y)=\inf\;\int_{0}^{1}|\dot{\gamma }|(t)\;dt,$$

where the infimum is taken over all absolutely continuous curves γ:[0,1]→X with γ(0)=x and γ(1)=y . If this infimum is obtained by a minimal curve, also called geodesic, we say that (X,d) is a geodesic space. As it turns out, the minimal curves obtained in theorem 2.2 are such geodesics. This can be immediately deduced from equation (A 9) and the definition of the metric velocity,

**Corollary 2.5.** *The space* $\left(P(M),W_{m,2}\right)$ *is a geodesic space*.

### (c) Gradient flows of the interaction energy

Let $W(x,y)\in C^{1}(M\times M)$ be a symmetric interaction kernel. The interaction energy is given by


$$\begin{array}{r} E(\mu )≔\frac{1}{2}\int_{M\times M}W(x,y)\;d\mu (x)\;d\mu (y). \end{array}$$

Let us consider the following inverse duality map:


$$J_{2}:x\in TM_{p}^{*}\mapsto |x{|}_{*}\underset{y\in TM_{p}:|y|=1}{\text{arg max}}x(y).$$

Since all tangent spaces are finite-dimensional, this map is well defined. The application of $J_{2}$ to a 1-form on M (in particular, a differential of a function) yields a velocity field on M . Below we show that gradient flows of the energy E with respect to the metric $W_{m,2}$ are given by weak solutions to PDEs of the form


(2.6)∂tμ+div(1mμJ2(DW[μ])μ)=0,

where $W[\mu ](x)=\int_{M}W(x,y)\;d\mu (y)$ . For M=S , $K(x,y)=e^{x\cdot Dy}$ and $W(x,y)=\pm e^{x\cdot Dy}$ equation (2.6) corresponds precisely to equation (2.1) if V=±D . The sole difference between equation (2.6) and classical Wasserstein gradient flows is the presence of the factor $\frac{1}{m_{\mu }}$ . It arises since the modified transport distance punishes the movement of particles with a high mobility $m_{\mu }(x)$ . When we interpret K(x,y) as an interaction kernel between particles, those particles interacting strongly with others are slowed down, while particles with low interaction are sped up.

**Lemma 2.6** (Chain rule). *Let* $t\to \mu_{t}$ *be an absolutely continuous curve in* $W_{2,m}$ . *Then* $t\mapsto E(\mu_{t})$ *is absolutely continuous and*


(2.7)ddtE(μt)=∫M⟨DW[μt](x),vt(x)⟩dμt(x)fora.et∈(0,T).

*Proof.* Let us consider an absolutely continuous curve (μ,v)∈CE(0,1;μ→ν) and the function $\eta :(x,t)\in M\times [0,T]\mapsto \frac{1}{2}\int_{M}W(x,y)\;d\mu_{t}(y)$ . In the case when $\eta \in C^{1}(M\times [0,T])$ , we could use it as a test function in equation (A 3) and immediately obtain


$$\begin{array}{rl} E(\mu_{T})-E(\mu_{0}) & =\int_{M}\eta (x,T)\;d\mu_{T}(x)-\int_{M}\eta (x,0)\;d\mu_{0}(x)\\ & =\int_{0}^{T}\int_{M}\partial_{t}\eta (x,t)\;d\mu_{t}(x)+\int_{M}\langle D\eta (t,x),v_{t}(x)\rangle \;d\mu_{t}(x)\;dt\\ & =\int_{0}^{T}\int_{M}\int_{M}\langle D_{x}W(x,y),v_{t}(x)\rangle \;d\mu_{t}(y)\;d\mu_{t}(x)\;dt<+\infty . \end{array}$$

The finiteness follows from the fact that we can bound $|D_{x}W(x,y){|}_{*}$ uniformly on M×M . In the general case, we have to use a rather lengthy time mollification argument, see appendix A(d).∎

Equation (2.7) is reminiscent of the classical chain rule $\frac{\;d}{\;dt}F(x(t))=\nabla F(x(t))\cdot \dot{x}(t)$ for a function $F:{\mathbb{R}}^{d}\to \mathbb{R}$ and a curve $x:[0,T]\to {\mathbb{R}}^{d}$ . The velocity field $v_{t}$ can be viewed as the ‘derivative’ of the curve $\mu_{t}$ , while $DW[\mu_{t}]$ is the corresponding ‘gradient’ of the interaction energy. Using this chain rule, we can estimate how fast the energy can decrease along a curve $\mu_{t}$ . Therefore, curves reaching this bound dissipate the energy as fast as possible and satisfy the so-called energy dissipation equality.

**Lemma 2.7.** *For any absolutely continuous w.r.t*. $W_{2,m}$ *curve* $(\mu_{t}{)}_{t\in (0,T)}$ , *we have that*


(2.8) $$\begin{array}{lrr} & E(\mu_{T})-E(\mu_{0})+\frac{1}{2}\int_{0}^{T}\int_{M}m_{\mu_{t}}|v_{t}{|}^{2}\;d\mu_{t}\;dt+\frac{1}{2}\int_{0}^{T}\int_{M}\frac{1}{m_{\mu_{t}}}|DW[\mu_{t}]{|}_{*}^{2}\;d\mu_{t}\;dt⩾0. & \end{array}$$

*Moreover, we have equality if and only if* $(\mu_{t}{)}_{t\in (0,T)}$ *is a weak solution to* *equation (2.6)*.

*Proof.* We can estimate the right-hand side of equation (2.7) by Hölder’s and Young’s inequalities:


$$\begin{array}{rl} \int_{M}DW[\mu_{t}](v_{t})\;d\mu_{t} & ⩾-\sqrt{\int_{M}m_{\mu_{t}}|v_{t}{|}^{2}\;d\mu_{t}}\sqrt{\int_{M}\frac{1}{m_{\mu_{t}}}|DW[\mu_{t}]{|}_{*}^{2}\;d\mu_{t}}\\ & ⩾-\frac{1}{2}\int_{M}m_{\mu_{t}}|v_{t}{|}^{2}\;d\mu_{t}-\frac{1}{2}\int_{M}\frac{1}{m_{\mu_{t}}}|DW[\mu_{t}]{|}_{*}^{2}\;d\mu_{t}. \end{array}$$

Integrating both sides of equation (2.7) from 0 to T, we obtain equation (2.8). Moreover, equality holds if and only if for a.e. t and $\mu_{t}$ -a.e. we have $v_{t}=\frac{1}{m_{\mu_{t}}}J_{2}(DW[\mu_{t}]$ ). Hence, $\mu_{t}$ is a weak solution to equation (2.6).∎

### (d) Metric gradient flows

Let us put the previous calculations into the context of curves of maximal slope [53, ch. 1], which can be viewed as a way to generalize gradient flows to general metric spaces. We assume (X,d) to be a complete metric space. Let E:X→ℝ . A function g:X→[0,+∞] is called a strong upper gradient of E if for any absolutely continuous curve x:[0,T]→X the concatenation g∘x is Borel and


$$\begin{array}{r} |E(x(t))-E(x(s))|⩽\int_{s}^{t}g(x(r))|\dot{x}|(r)\;dr\;\forall 0⩽s⩽t⩽T. \end{array}$$

If E(x(t)) is non-increasing in t then the application of Young’s inequality yields


$$\begin{array}{r} E(x(t))-E(x(s))+\frac{1}{2}\int_{s}^{t}g(x(r){)}^{2}+|\dot{x}|(r{)}^{2}\;dr⩾0\;\forall 0⩽s⩽t⩽T. \end{array}$$

This observation allows us to define curves of maximal slope as those that decrease the energy as fast as possible.

**Definition 2.8** (Curve of maximal slope). An absolutely continuous curve x:[0,T]→X is called a curve of maximal slope of E with respect to its strong upper gradient g if t↦E(x(t)) is non-increasing and


$$\begin{array}{r} E(x(t))-E(x(s))+\frac{1}{2}\int_{s}^{t}g(x(r){)}^{2}+|\dot{x}|(r{)}^{2}\;dr⩽0\;\forall 0⩽s⩽t⩽T. \end{array}$$

**Lemma 2.9.** *The map*


$$\begin{array}{r} g:\mu \mapsto \sqrt{\int_{M}\frac{1}{m_{\mu }}|DW[\mu_{t}]{|}_{*}^{2}\;d\mu } \end{array}$$

*is a strong upper gradient of* E *and solutions of* equation (2.6) *coincide with curves of maximal slope of* E *with respect to the strong upper gradient* g .

*Proof.* For an absolutely continuous w.r.t. $W_{2,m}$ curve $\mu_{t}$ , we can find, by lemma 2.4, a velocity field $(v_{t}{)}_{t\in (0,T)}$ such that (μ,v)∈CE(0,T) and


$${\left(\int_{M}m_{\mu_{t}}|v_{t}{|}^{2}\;d\mu_{t}\right)}^{1/2}=|\dot{\mu }|(t)\;\text{for a.e. }t\in (0,T).$$

Then, the chain rule, lemma 2.6 yields


$$\begin{array}{r} |E(\mu_{t})-E(\mu_{s})|⩽\int_{s}^{t}|\langle DW[\mu_{t}],v_{r}\rangle |\;dr⩽\int_{s}^{t}g(\mu_{r})|\dot{\mu }|(r)\;dr, \end{array}$$

and g is a strong upper gradient. The coincidence of solutions of equation (2.6) and curves of maximal slope follows from lemma 2.7.∎

### (e) Energy dissipation and large-time behaviour

Due to the missing geodesic convexity properties of the energy, we cannot expect convergence of the evolution to a unique minimizer in the large time limit. However, we can obtain some weaker results by further analysing the energy dissipation property:


(2.9) $$\begin{array}{lcr} & E(\mu_{t})+\frac{1}{2}\int_{0}^{t}\int_{M}m_{\mu_{s}}(x)|\nabla E^{\prime }(\mu_{s}){|}^{2}\;d\mu_{s}(x)\;\;ds⩽E(\mu_{0}). & \end{array}$$

As s→∞ , we can pick narrowly convergent subsequences of $\mu_{s}$ (i.e. converging weakly star in the Banach space of Radon measures). Moreover, the entropy dissipation inequality above implies


$$\int_{0}^{\infty }\int_{M}m_{\mu_{s}}(x)|\nabla E^{\prime }(\mu_{s}){|}^{2}\;\;d\mu {,}_{s}(x)\;\;ds<\infty ,$$

hence, along suitable subsequences, the entropy dissipation,


$$D(s)=\int_{M}m_{\mu_{s}}(x)|\nabla E^{\prime }(\mu_{s}){|}^{2}\;d\mu_{s}(x),$$

converges to zero since it is non-negative and bounded. To establish the existence of subsequences converging to stationary solutions, we need to identify the limit in suitable spaces. Under appropriate regularity assumptions on the interaction kernel W (satisfied, for example, for the exponential kernel), this is a direct consequence of the Arzelà–Ascoli theorem.

**Lemma 2.10.** *Let* M *be a compact manifold without a boundary,* $W\in C^{1,\alpha }(M\times M)$ *for some* α>0 *and symmetric. Moreover, let* $\mu^{n}$ *be a sequence of probability measures on* M . *Then the sequences*


$$m_{\mu^{n}}=\int_{M}W(\cdot ,y)\;d\mu^{n}(y)\;\text{and}\;\nabla E^{\prime }(\mu^{n})=\int_{M}\nabla_{x}W(\cdot ,y)\;d\mu^{n}(y)$$

*have uniformly convergent subsequences. If* $\mu^{n}$ *converges narrowly to* $\mu^{*}$ *, then* $m_{\mu^{n}}$ *converges uniformly to* $m_{\mu^{*}}$ *and* $\nabla E^{\prime }(\mu^{n})$ *converges uniformly to* $\nabla E^{\prime }(\mu^{*}).$

Lemma 2.10 combined with the entropy dissipation inequality (2.9) yields the following result.

**Corollary 2.11.** *Let* M *be a compact manifold without a boundary,* $W\in C^{1,\alpha }(M\times M)$ *for some* α>0 *and symmetric. Then each weak solution* $\mu_{t}$ *of* equation (2.1) *with the velocity field given by* equation (1.8) *has a narrowly convergent subsequence* $\mu_{t_{n}}$ *as* $t_{n}\to \infty$ , *the limit of which is a stationary solution*.

The following example connects the general results of this section with the transformer dynamics.

**Example 2.12**. The transformer dynamics for a finite number of particles described by equation (1.7) with V=±D correspond to the choice M=S , $K(x,y)=e^{x\cdot Dy}$ and $W(x,y)=\pm e^{x\cdot Dy}$ . As discussed in §1d, the corresponding empirical measures $\mu_{t}$ fulfil the continuity equation (1.9). Thus, they solve equation (2.1) in the weak sense with the velocity field given by equation (1.8), and all requirements of corollary 2.11 are fulfilled. Therefore, there exists a subsequence of $\mu_{t}$ that converges narrowly to a stationary solution of the interaction energy $E_{D}$ defined in equation (1.2).

This section establishes the relation between the particle model in equation (1.7) and gradient flows of interaction energies for the special cases V=±D . The energy dissipation property equation (2.8) and convergence property from corollary 2.11 motivate the study of stationary solutions of the energy $E_{D}$ , which we carry out in §§3 and 4. We shall start with minimizers and maximizers.

## 3. Explicit energy minimizers and maximizers

In this section, we compute explicit minimizers and maximizers of the energy $E_{D}$ (from equation (1.2), i.e. without the factor 1/2 ) in different scenarios, depending on the properties of the interaction matrix D . We make the dependence on the matrix D explicit by employing it as a subscript of the energy. The case D=Id⁡ has already been covered in [9, Proposition 3.4], where it is stated that a measure is a maximizer if and only if it is a Dirac delta placed at any point on the sphere, and a minimizer if and only if it is the uniform distribution. As we show below, for more general matrices, the position of optimal Diracs depends strongly on the eigenvalues of the matrix D . We further derive a symmetry condition for minimizers of energies with a positive definite interaction matrix D . This property yields an alternative, simpler proof that the uniform distribution is the only minimizer for D=Id⁡ .

### (a) Maximal eigenvalue and related maximizers or minimizers

Like for D=Id⁡ , there are several cases in which the minimizers or maximizers of the energy $E_{D}$ are given by Diracs concentrated at a single point. We start with the maximizers when the largest eigenvalue of D is also an eigenvalue of the largest absolute value (or, respectively, minimizers when the smallest eigenvalue of D is also an eigenvalue of the largest absolute value).

**Theorem 3.1.** *Let* λ *be an eigenvalue of maximal absolute value of* D *and* $Z_{\lambda }\subseteq S$ *the set of associated normalized eigenvectors. If* λ>0 *then* $\mu^{*}=\delta_{z}$ *with* $z\in Z_{\lambda }$ *are the only maximizers of the energy* $E_{D}$ . *If* λ<0 *then* $\mu^{*}=\delta_{z}$ *with* $z\in Z_{\lambda }$ *are the only minimizers*.

*Proof.* We consider the case λ>0 ; the case λ<0 can be treated similarly. For all x,y∈S , we have $e^{x\cdot Dy}⩽e^{\lambda }$ with equality if and only if x=y=±z . Thus,


$$E_{D}(\mu )=\int_{S}\int_{S}e^{x\cdot Dy}\;d\mu (x)\;d\mu (y)⩽\int_{S}\int_{S}e^{\lambda }\;d\mu (x)\;d\mu (y)=e^{\lambda }=E_{D}(\mu^{*}),$$

where the inequality is strict if μ is not concentrated on an eigenvector associated with λ .∎

An example of the above setting is maximizing the energy for D=Id⁡ [9, Proposition 3.4], where the authors make a connection between the existence of concentrated maximizers and the so-called mode collapse of transformers often observed in practice. For a positive definite D≠Id⁡ , theorem 3.1 shows that the set of maximizers is not only restricted to Dirac measures, but that it is actually finite. We summarize this insight in the following example and refer to §5a for an illustrating numerical example.

**Example 3.2.** If D=Id⁡ then $\mu^{*}=\delta_{z}$ is a maximizer of the energy $E_{\mathrm{Id}}$ for any z∈S . Similarly, for D=−Id⁡ , $\mu^{*}=\delta_{z}$ is a minimizer for any z∈S . If D≠Id⁡ is positive definite then $\mu^{*}=\delta_{z}$ is a maximizer of $E_{D}$ only if Dz=λz and λ is the largest eigenvalue of D . Similarly, for a negative definite D≠Id⁡ , $\mu^{*}=\delta_{z}$ is a minimizer only if Dz=λz and λ is the smallest eigenvalue of D .

In the remainder of this section, we study minimizers for matrices that do not fulfil the conditions of theorem 3.1.

### (b) Minimizers for indefinite matrices

We now generalize the statement in theorem 3.1 to minimizers of energies where the matrix D has at least one non-positive eigenvalue. In particular, we do not assume that the smallest eigenvalue is the eigenvalue of maximal absolute value. A key property is the following result that gives a lower bound on the energy in terms of the smallest eigenvalue of D .

**Lemma 3.3.** *Let* $\bar{x}$ *be the expected value of* x *under* μ *, i.e.* $\bar{x}:=\int_{S}x\;d\mu (x)$ . *Then*


(3.1) $$\begin{array}{lcr} & E_{D}(\mu )⩾e^{\bar{x}\cdot D\bar{x}}. & \end{array}$$

*If* D *is not positive definite and* $\lambda {,}_{\min}$ *is its smallest eigenvalue, it further holds that*


(3.2)ED(μ)⩾eλmin.

*Proof.* We use the convexity of exponential functions of the form $x\mapsto e^{x\cdot a}$ and $y\mapsto e^{b\cdot y}$ for arbitrary $a,b\in {\mathbb{R}}^{n}$ *,* which, with two applications of Jensen’s inequality, implies


(3.3) $$\begin{array}{lrlr} & E_{D}(\mu ) & =\int_{S}\int_{S}e^{x\cdot Dy}\;d\mu (y)\;d\mu (y)\int_{S}e^{x\cdot D\bar{x}}\;d\mu (x)⩾e^{\bar{x}\cdot D\bar{x}}. & \end{array}$$

Since, further, $\bar{x}\cdot D\bar{x}⩾\lambda_{\min}\;\|\bar{x}{\|}^{2}$ and $0⩽\|\bar{x}\|⩽1$ , the monotonicity of the exponential function gives us


$$E_{D}(\mu )⩾e^{\min\left\{\lambda_{\min},0\right\}}.$$

If D is not positive definite, we know that $\lambda {,}_{\min}⩽0$ and the above inequality reduces to inequality (3.2).∎

A direct consequence of lemma 3.3 for indefinite matrices is that a Dirac measure that is concentrated on an eigenvector corresponding to the smallest eigenvalue is a minimizer of the energy. If the smallest eigenvalue is negative, we can even show that all minimizers are of this form. In the case of a vanishing smallest eigenvalue, it is necessary and sufficient that the measure is concentrated on the null space of D .

**Theorem 3.4.** *Consider a matrix* D *that is not positive definite with the smallest eigenvalue* $\lambda {,}_{\min}⩽0$ . *If* $\lambda {,}_{\min}<0$ *, a measure minimizes the energy if and only if it is a Dirac measure placed at an eigenvector corresponding to* $\lambda {,}_{\min}$ . *If* $\lambda {,}_{\min}=0$ *, a measure minimizes the energy if and only if it is concentrated on the null space of* D .

*Proof.* We first assume $\lambda {,}_{\min}<0$ . It follows directly from equation (3.2) that every Dirac measure concentrated on an eigenvector corresponding to $\lambda {,}_{\min}$ is a minimizer. We further see that $\bar{x}\cdot D\bar{x}=\lambda {,}_{\min}$ if only if $\bar{x}$ is an eigenvector corresponding to $\lambda {,}_{\min}$ and $\|\bar{x}\|=1$ . This can only hold for Dirac measures. Thus, there are no other minimizers.

For $\lambda {,}_{\min}=0$ , it also follows directly from equation (3.2) that every measure concentrated on the null space of D minimizes the energy. However, $\bar{x}\cdot D\bar{x}=\lambda {,}_{\min}$ holds for all measures that fulfil $\bar{x}=0$ . Still, the estimate in equation (3.3), obtained using Jensen’s inequality, is only an equality if $x\cdot Dx=\bar{x}\cdot D\bar{x}=0$ for μ -a.e. x∈S . Therefore, all minimizers are concentrated on the null space of D .∎

**Remark 3.5.** *In general, theorem 3.4 does not transfer to maximizers for matrices* D *that are not negative definite. To see this, consider* D *with the largest eigenvalue* $\lambda {,}_{\max}⩾0$ *, the smallest eigenvalue* $\lambda {,}_{\min}<0$ *and corresponding eigenvectors* $z_{\min}$ *and* $z_{\max}$ . *If further* $e^{\lambda_{\max}}<\cosh(\lambda_{\min})$ , *it holds that*


$$E_{D}\left(\delta_{z_{\max}}\right)=e^{\lambda_{\max}}<\cosh\left(\lambda_{\min}\right)=E_{D}\left(\frac{\delta_{z_{\min}}+\delta_{-z_{\min}}}{2}\right)$$

*and thus,* $\delta_{z_{\max}}$ *is not a maximizer. In the special case* $\lambda {,}_{\max}=0$ *, the above inequality holds for all measures concentrated on the null space of* D *and all* $\lambda {,}_{\min}<0$ .

At this point, we further note that the above strategy does not work for analysing minimizers for positive definite interaction matrices D . In this case, lemma 3.3 not only gives us $E_{D}(\mu )⩾e^{0}=1$ , but also x⋅Dx>0 for all x∈S , so the inequality is strict for all measures μ∈P(S) .

### (c) Symmetry property for positive definite matrices

The remainder of this section gives the first characterization of minimizers of the energy when the interaction matrix is positive definite. More precisely, we can show that, in this case, all minimizers are symmetric, and the symmetry axes are determined by the eigenvectors of D . The first step towards this is to show that the energy $E_{D}$ is strictly convex if D is positive definite.

**Lemma 3.6.** *If* D *is positive semi-definite (resp. positive definite) then* $E_{D}$ *is convex (resp. strictly convex*).

*Proof.* Since $E_{D}$ is quadratic, convexity (resp. strict convexity) follows from the non-negativity (resp. positivity) of the quadratic form:


$$F(\mu )=\int_{S}\int_{S}e^{x\cdot Dy}\;d\mu (x)\;d\mu (y),$$

for arbitrary signed Radon measures μ , e.g. [56, Proposition 2.11]. For D positive semi-definite, there exists a unique positive semi-definite matrix square root $D^{1/2}$ and we can use the transformation $T(x)=D^{1/2}x$ . We denote by $T_{\#}\mu$ the pushforward of μ by T , so that


$$\begin{array}{rl} F(\mu ) & =\int_{T(S)}\int_{T(S)}e^{x\cdot y}\;dT_{\#}\mu (x)\;dT_{\#}\mu (y)\\ & =\int_{T(S)}\int_{T(S)}e^{-\frac{1}{2}|x-y{|}^{2}}e^{\frac{1}{2}|x{|}^{2}}\;dT_{\#}\mu (x)e^{\frac{1}{2}|y{|}^{2}}\;dT_{\#}\mu (y). \end{array}$$

Let $\;d\eta =e^{\frac{1}{2}|x{|}^{2}}\;dT_{\#}\mu (x)$ , then


$$F(\mu )=\int_{T(S)}\int_{T(S)}e^{-\frac{1}{2}|x-y{|}^{2}}\;d\eta (x)\;d\eta (y).$$

The fact that the Gaussian kernel is positive definite (e.g. [57]) yields that F(μ)>0 unless ν vanishes. This can only happen if μ=0 or, in the case of a semi-definite matrix D , if μ is concentrated on the null space N(D) and μ(N(D))=0 . This yields the assertion.∎

**Remark 3.7.** *The previous convexity result does not guarantee the convergence of the gradient flow in (* *equation (2.6)* *) to a global minimizer of* F . *For such results, usually a slightly different notion of convexity is required, the so-called geodesic convexity. The following example shows that besides the case of* D *being a multiple of the identity, we do not have geodesic convexity for the classical* 2 *-Wasserstein distance. We do not expect any improvements for our modified optimal transport distance*.

**Example 3.8.** We consider a simple counterexample in $S^{1}$ (equipped with the spherical distance) to show that F is not convex along 2 -Wasserstein geodesics. Choose


$$D=\left(\begin{array}{cc} 2 & 0\\ 0 & 1 \end{array}\right)\;\text{and the curve}\;\gamma :t\in [0,1]\mapsto \left(\begin{array}{c} \cos(-\frac{\pi }{4}+t\frac{\pi }{2})\\ \sin(-\frac{\pi }{4}+t\frac{\pi }{2}) \end{array}\right).$$

Then $\mu_{t}≔\delta_{\gamma (t)}$ is a constant-speed geodesic in the 2 -Wasserstein space connecting $\delta_{\gamma (0)}$ and $\delta_{\gamma (1)}$ . Clearly, the map [0,1]∋t↦F(γ(t)) is not convex, since


$$F(\gamma (0))=F(\gamma (1))=e^{1.5}<e^{2}=F\left(\gamma \left(\frac{1}{2}\right)\right).$$

Such a counterexample can always be constructed as long as D has two different eigenvectors. Lemma 3.6 does not contradict this counterexample, however, as it only implies the convexity of


$$[0,1]∋t\mapsto F((1-t)\mu_{0}+t\mu_{1}).$$

Having established convexity, we can show that reflecting a measure along the eigenvectors of D and then normalizing it does not increase the energy. Moreover, if D is positive definite and μ is not symmetric with respect to all eigenvectors of D , one can always construct a symmetric measure with a smaller energy.

**Lemma 3.9.** *Let* z *be an eigenvector related to an eigenvalue* λ *of a positive semi-definite matrix* D . *For a measure* μ *, we define* $\tilde{\mu }$ *as*


$$\begin{array}{r} \tilde{\mu }:=\frac{1}{2}\left(\mu +{H_{z}}_{\#}\mu \right),\;H_{z}(x)=x-2(x\cdot z)z, \end{array}$$

*where* $H_{z}$ *denotes a reflection. Then,* $E_{D}(\tilde{\mu })⩽E_{D}(\mu )$ *and the inequality is strict if* D *is positive definite and* $\tilde{\mu }\neq \mu$ .

*Proof*. Since $e^{x\cdot Dy}=e^{H_{z}(x)\cdot DH_{z}(y)}$ , it is straightforward to see that $E_{D}(\mu )=E_{D}({H_{z}}_{\#}\mu )$ . The (strict) convexity of the energy yields the assertion.∎

As a direct consequence, we obtain a symmetry property of minimizers for positive definite D .

**Corollary 3.10.** *If* D *is positive definite then each minimizer is symmetric with respect to its eigenvectors*.

If D is a positive multiple of the identity, one can easily show using the above result that the uniform distribution is the unique energy minimizer. This has been shown already in [9, Proposition 3.4] using properties of Gegenbauer polynomials [58, Proposition 2.2]. The symmetry property from corollary 3.10 gives an alternative—and straightforward—proof of this fact.

**Proposition 3.11.** *If* D=λId⁡ *for* λ>0 *then the uniform distribution is the unique energy minimizer*.

*Proof.* If μ is not uniform, we can find a unit vector z such that with $H_{z}$ as in lemma 3.9, we have


$$\tilde{\mu }=\frac{1}{2}\left(\mu +{H_{z}}_{\#}\mu \right)\neq \mu .$$

However, for D=λId⁡ , every unit vector is an eigenvector and lemma 3.9 implies that $E_{D}(\tilde{\mu })<E_{D}(\mu )$ . Hence, the uniform distribution is the only minimizer of the energy.∎

**Remark 3.12.** *The statement in proposition 3.11 does not transfer to maximizers for negative multiples of the identity. To see this, consider* D=λId⁡ *with* λ<0 *and let* $\mu_{0}$ *denote the uniform distribution on* S . *The symmetry of* $\mu_{0}$ *yields*


$$E_{D}\left(\mu_{0}\right)=2\int_{S^{+}}\int_{S^{+}}e^{\lambda x\cdot y}+e^{-\lambda x\cdot y}\;d\mu_{0}(x)d\mu_{0}(y)=4\int_{S^{+}}\int_{S^{+}}\cosh(\lambda x\cdot y)d\mu_{0}(x)d\mu_{0}(y),$$

*where* $S^{+}:=\left\{x\in S:\;x{,}_{1}>0\right\}$ . *Since* |x⋅y|<1 $\mu_{0}\times \mu_{0}$ *-almost everywhere on* $S^{+}\times S^{+}$ *the integrand can be strictly bounded from above by* 4cosh⁡(λ) . *Since* $\mu_{0}(S^{+})=1/2$ *it follows that*


$$\begin{array}{r} E_{D}(\mu_{0})<\cosh(\lambda )=E_{D}\left(1/2\;(\delta_{z}+\delta_{-z})\right), \end{array}$$

*with* z∈S . Therefore, $\mu_{0}$ cannot be a maximizer of $E_{D}$ .

**Remark 3.13.** *The above argument can be used to show that for arbitrary* D *, one has*


$$\begin{array}{r} E_{D}(\mu )⩽E_{D}\left(\frac{\delta_{z}+\delta_{-z}}{2}\right) \end{array}$$

*for all symmetric measures* μ *if and only if* z *is an eigenvector that corresponds to the eigenvalue of the largest absolute value. In the upcoming section, we use this insight to show that such measures are maximizers of* $E_{D}$ *for negative semi-definite* D .

If D has non-positive eigenvalues, theorems 3.1 and 3.4 still show that all minimizers are invariant with respect to reflections $H_{z}$ , where z corresponds to a positive eigenvalue. However, if D has negative eigenvalues, such reflections can increase the energy when they are applied to general, non-minimizing measures. This is illustrated by the following example.

**Example 3.14.** Consider the two-dimensional case with D=diag(λ,1) and λ<0 . For any θ∈[0,2π) *,* denote by $\delta_{\theta }$ the Dirac delta placed at (cos⁡(θ),sin⁡(θ)) . Fix φ∈[0,2π) and let


$$\begin{array}{r} \mu =\frac{1}{2}(\delta_{\varphi }+\delta_{\pi +\varphi }). \end{array}$$

In the two-dimensional setting, the symmetrization is given by


$$\begin{array}{r} \tilde{\mu }=\frac{1}{4}(\delta_{\varphi }+\delta_{\pi +\varphi }+\delta_{-\varphi }+\delta_{\pi -\varphi }). \end{array}$$

Denoting, for convenience, cos⁡(φ)=c , we have


$$E_{D}(\mu )-E_{D}(\tilde{\mu })=\frac{1}{2}(\cosh\left|(\lambda -1)c^{2}+1\right|-\cosh\left|(-\lambda -1)c^{2}+1\right|).$$

Since t↦cosh⁡(t) is strictly increasing for t⩾0 , we get that $E_{D}(\mu )⩽E_{D}(\tilde{\mu })$ since


$$\left|(\lambda -1)c^{2}+1\right|=\left|-|\lambda |c^{2}+1-c^{2}\right|⩽|\lambda |c^{2}+1-c^{2}=\left||\lambda |c^{2}+1-c^{2}\right|=\left|(-\lambda -1)c^{2}+1\right|$$

for any 0⩽c⩽1 and λ⩽0 , and the inequality is strict if and only if 0<c<1 and λ<0 .

### (d) Maximizers for negative semi-definite matrices

There is no apparent way to use the proof strategy from the previous section for showing that maximizers for negative definite matrices are symmetric, since the kernel $(x,y)\mapsto e^{x\cdot Dy}$ is not negative definite for a negative definite D . However, we can show that the quadratic form F used to prove lemma 3.6 is non-positive for anti-symmetric measures. This yields a symmetry property of maximizers for negative semi-definite matrices.

**Lemma 3.15.** *Let* D *be a negative semi-definite matrix and* μ *a measure on the sphere. Define* $\tilde{\mu }$ *as*


$$\begin{array}{r} \;d\tilde{\mu }(x)=\frac{1}{2}(d\mu (x)+\;d\mu (-x)). \end{array}$$

*Then* $E_{D}\left(\tilde{\mu }\right)⩾E_{D}\left(\mu \right)$ *and the inequality is strict if* $\tilde{\mu }\neq \mu$ *and either* D *is negative definite or* $\tilde{\mu }=\mu$ *on the null space* N(D) .

*Proof.* We denote by N(x)=−x the negation and define


$$\begin{array}{r} \mu^{+}:=\mu ,\;\mu^{-}:=N_{\#}\mu ,\;\zeta :=1/2\;(\mu^{-}-\mu^{+}). \end{array}$$

This yields that dζ(−x)=2(dμ(−x)−dμ(x))=−dζ(x) and


$$\begin{array}{rl} E_{D}(\zeta )= & \int_{S}\int_{S}e^{x\cdot Dy}\;d\zeta (x)\;d\zeta (y)=\int_{S^{+}}\int_{S^{+}}e^{x\cdot Dy}\;d\zeta (x)\;d\zeta (y)+\int_{S^{+}}\int_{S^{+}}e^{x\cdot Dy}\;d\zeta (-x)\;d\zeta (-y)\\ & +2\int_{S^{+}}\int_{S^{+}}e^{-x\cdot Dy}\;d\zeta (-x)\;d\zeta (y)=2\int_{S^{+}}\int_{S^{+}}e^{x\cdot Dy}-e^{-x\cdot Dy}\;d\zeta (x)\;d\zeta (y)=-E_{-D}(\zeta ). \end{array}$$

Since −D is positive semi-definite, the proof of lemma 3.6 shows that $E_{-D}(\zeta )⩾0$ and thus $E_{D}(\zeta )⩽0$ . The inequality is strict if ζ≠0 and either D is negative definite or ζ is concentrated on $N(D{)}^{⟂}$ . The symmetry of the kernel yields $E_{D}(\mu^{-})=E_{D}(\mu^{+})$ . Further, by substituting $\mu^{+}=\tilde{\mu }+\zeta$ and $\mu^{-}=\tilde{\mu }-\zeta$ , we see that


$$\begin{array}{rl} E_{D}(\tilde{\mu }) & =\frac{1}{4}E_{D}\left(\mu^{+}\right)+\frac{1}{4}E_{D}\left(\mu^{-}\right)+\frac{1}{2}E_{D}\left(\mu^{+},\mu^{-}\right)\\ & =\frac{1}{2}E_{D}(\mu )+\frac{1}{2}E_{D}(\tilde{\mu }+\zeta ,\tilde{\mu }-\zeta )=\frac{1}{2}E_{D}(\mu )+\frac{1}{2}E_{D}(\tilde{\mu })-\frac{1}{2}E_{D}(\zeta ). \end{array}$$

Reordering the terms leads to


$$E_{D}(\tilde{\mu })=E_{D}(\mu )-E_{D}(\zeta )⩾E_{D}(\mu ).$$

From the conditions on ζ and D that lead to $E_{D}<0$ , we derive that the above inequality is strict if $\tilde{\mu }\neq \mu$ and either D negative definite or $\tilde{\mu }=\mu$ on N .∎

**Corollary 3.16.** *Let* $\mu^{*}$ *be a maximizer of* $E_{D}$ *for a negative definite* D . *Then* $\;d\mu^{*}(x)=\;d\mu^{*}(-x)$ .

This symmetry property is the missing ingredient for showing that the discrete measures introduced in remarks 3.12 and 3.13 are maximizers for negative semi-definite matrices D .

**Theorem 3.17.** *Let* D *be negative semi-definite and* $\lambda {,}_{\min}<0$ *its smallest eigenvalue. Then, a measure* μ *maximizes* $E_{D}$ *if and only if* $\mu^{*}=1/2\;(\delta_{z}+\delta_{-z})$ *where* z∈S *is an eigenvector associated with* $\lambda {,}_{\min}$ .

*Proof.* By lemma 3.15, it suffices to consider μ satisfying dμ(x)=dμ(−x) . Denoting $S^{+}:=\left\{x\in S:x_{1}>0\right\}$ and using the symmetry property of μ , with the arguments from remark 3.12, we have


$$E_{D}(\mu )⩽\cosh\;\lambda_{\min}=E_{D}\left(\mu^{*}\right),$$

where equality is only obtained if $|x\cdot Dy|=\lambda {,}_{\min}$ holds μ×μ -almost everywhere on $S^{+}\times S^{+}$ . Since μ is symmetric, this is equivalent to $\mu =\mu^{*}$ . For a negative definite D , we already know from corollary 3.16 that there are no other measures that maximize $E_{D}$ . In the negative semi-definite case, we have that any μ that fulfils $E_{D}\left(\mu \right)=\cosh\lambda_{\text{min}}$ has to be concentrated on $N(D{)}^{⟂}$ and, therefore, also in this case, there are no other maximizers.∎

## 4. Energy variation and stationary points

To study stationary points or local maximizers/minimizers, it is useful to consider the first and second variations of the energy on the Wasserstein space of probability measures on the sphere, as studied previously for Vlasov-type interactions, e.g. the mean-field aggregation equation, cf. [36,59,60]. The first variation of $E_{D}$ is given by


(4.1) $$\;dE_{D}(\mu ;V)=\frac{\;d}{\;dt}E_{D}(\mu_{t}){|}_{t=0},$$

where $\mu_{t}$ satisfies


(4.2) $$\begin{array}{lcr} & \partial_{t}\mu_{t}+\nabla \cdot (\mu_{t}P_{x}^{⟂}V)=0,\;\mu_{0}=\mu , & \end{array}$$

and $P_{x}^{⟂}=\mathrm{Id}-xx^{T}$ is the projection to the tangent space of the unit ball at x . Here, the velocity field V is an arbitrary Lipschitz function on ${\mathbb{R}}^{n}$ ; by the projection $P_{x}^{⟂}$ , we restrict it further to admissible velocities that keep the distribution on the unit sphere.

The following weak formulation, where φ is a continuously differentiable test function, will be useful later:


$$\frac{\;d}{\;dt}\int_{S}\varphi (x)\;\;d\mu {,}_{t}(x)=\int_{S}P_{x}^{⟂}\nabla \varphi (x)\cdot V(x)\;\;d\mu {,}_{t}(x).$$

Similar to the first variation, the second variation of $E_{D}$ can be defined as


(4.3) $$\begin{array}{lcr} & \;d{,}^{2}E_{D}(\mu ;V.W)=\frac{\;d}{\;dt}\;dE_{D}(\mu_{t},W){|}_{t=0} & \end{array}$$

if the derivative on the right-hand side exists. The computation of the first variation is completely analogous to the case of the aggregation equation (cf. [59]) and thus omitted here.

**Lemma 4.1.** *For any Lipschitz continuous vector field* V *, the first variation of the energy* $E_{D}$ *in the direction* V *exists and is given by*


(4.4) $$\begin{array}{lcr} & \;dE_{D}(\mu ;V)=\int_{S}\int_{S}e^{x\cdot (Dy)}P_{x}^{⟂}Dy\cdot V(x)\;d\mu (x)\;d\mu (y). & \end{array}$$

It is straightforward to see that the first variation vanishes at the extremal points of the energy:

**Proposition 4.2.** *Let* $\mu^{*}$ *be a minimizer or maximizer of the energy. Then* $\;dE_{D}(\mu ;V)=0$ *for all Lipschitz vector fields* V .

*Proof.* Let $\mu^{*}$ be the initial value for the transport equation (4.2). For Lipschitz-continuous vector fields, there is a unique solution $\mu_{t}$ of the transport equation, and for all times t>0 , it is an admissible distribution on the sphere. Hence, if $\mu^{*}$ is a minimizer, then


$$E_{D}(\mu^{*})⩽E_{D}(\mu_{t})$$

for all t>0 , which implies that $\;dE_{D}(\mu^{*};V)⩽0$ in the limit t↓0 . Since V is arbitrary and $\;dE_{D}$ is linear in V , we have that $\;dE_{D}(\mu ;V)=0$ . The case of a maximizer is treated in the same way, with an opposite inequality initially.∎

The connection between the transformer dynamics and the energy variations in Wasserstein spaces is readily established in the following.

**Lemma 4.3.** *A probability measure* μ *is a stationary solution of* equation (2.1) *with the velocity field given by* equation (1.8) *if and only if* $\;dE_{D}(\mu ;W)=0$ *for all Lipschitz continuous* W .

Similarly to lemma 4.1, one can obtain an expression for the second variation.

**Lemma 4.4.** *For* V,W *being Lipschitz continuous, the second variation of the energy* $E_{D}$ *in the directions* V *,* W *exists and is given by*


$$\;dE_{D}(\mu ;V,W)=\int_{S}\int_{S}e^{x\cdot Dy}((P_{x}^{⊥}Dy\cdot V(x))(P_{x}^{⊥}Dy\cdot W(x))+(Dy{)}^{T}\nabla (P_{x}^{⊥}V(x)))\;d\mu (x)\;d\mu (y).$$

### (a) Energy variation at concentrated distributions

From lemma 4.1, we see that any measure μ that fulfils


(4.5) $$\begin{array}{lrr} & \int_{S}e^{x\cdot Dy}P_{x}^{⟂}Dy\;\;d\mu (y)=0\;\text{for }\mu \text{-almost all }x\in S, & \end{array}$$

is a stationary point of $E_{D}$ . Here and in the following, with a slight abuse of notation, we denote the 0 -vector by 0 . For concentrated measures, the above condition is also necessary and rather easy to verify, as we see in what follows. We first show that single Dirac measures can only be stationary points if they align with an eigenvector of the matrix D .

**Lemma 4.5.** *A Dirac measure* $\mu^{*}=\delta_{z}$ *is a stationary point of* $E_{D}$ *if and only if* z *is an eigenvector of* D .

*Proof.* The first variation is given by


$$\;dE_{D}(\mu^{*};V)=-e^{z\cdot Dz}P_{z}Dz\cdot V(z).$$

Since V(z) is an arbitrary vector, $\mu^{*}$ is a stationary point if and only if


$$0=P_{z}^{⟂}Dz=Dz-(z^{T}Dz)z,$$

which holds if and only if z is an eigenvector of D .∎

Intuitively speaking, $P_{z}^{⟂}Dz=0$ means that the force emerging from the interaction of a particle located at eigenvector z with itself is orthogonal to the tangent space of S at point z and is thus cancelled out by the projection. The same effect can be observed for convex combinations of a Dirac measure and its reflection.

**Lemma 4.6.** *For any* t∈[0,1] , *we have that* $t\delta_{z}+(1-t)\delta_{-z}$ *is a stationary point of* $E_{D}$ *if and only if* z *is an eigenvector of* D .

*Proof*. Using the expression in lemma 4.1, we obtain for any Lipschitz continuous V , using the abbreviation ι=z⋅Dz , that


$$\begin{array}{rl} \;dE_{D}(t\delta_{z}+(1-t)\delta_{-z};V)= & \;t^{2}\;e{\;}^{\iota }P_{z}^{⊥}Dz\;V(z)+(1-t{)}^{2}\;e{\;}^{\iota }P_{-z}^{⊥}D(-z)\;V(-z)\\ & +t(1-t)\;e{\;}^{-\iota }P_{-z}^{⊥}Dz\;V(z)+t(1-t)\;e{\;}^{-\iota }P_{z}^{⊥}D(-z)\;V(-z). \end{array}$$

We first observe that for any x,y one has that $P_{x}^{⟂}y=P_{-x}^{⟂}y=-P_{x}^{⟂}(-y)$ . By comparing the coefficients in the above equation, we obtain that


$$\begin{array}{rl} dE_{D}(t)\delta_{z} & +(1-t)\delta_{-z};V)=0\;\text{ for all }V\text{ Lipschitz }\;\Leftrightarrow \;P_{z}^{⊥}Dz=0\\ & \Leftrightarrow Dz-(z\cdot Dz)z=0\;\Leftrightarrow \;z\text{ is an eigenvector. } \end{array}$$

∎

For the symmetric case t=1/2 in the above lemma, we can further show that any convex combination of such stationary points is again a stationary point.

**Lemma 4.7.** *Let* $Z_{D}$ *be a finite subset of eigenvectors of* D *such that* w⋅z=0 *for all* $z\in Z_{D}\backslash \{w\}$ . *Then for any choice of parameters* $t:Z_{D}\to {\mathbb{R}}_{0}^{+}$ *such that* $\sum_{z\in Z_{D}}t(z)=1$ *the following measure is a stationary point of* $E_{D}$ :


$$\mu =\frac{1}{2}\sum_{z\in Z_{D}}t(z)\left(\delta_{z}+\delta_{-z}\right).$$

*Proof.* We prove the statement by showing that equation (4.5) holds. For any $w\in Z_{D}$ , it holds that


$$\begin{array}{r} P_{w}^{⟂}Dw=-P_{w}^{⟂}Dw=0, \end{array}$$

since $Z_{D}$ only contains eigenvectors of D . On the other hand, since we also require w⋅z=0 for all $z\in Z_{D}\backslash \{w\}$ it follows that z⋅Dw=−z⋅Dw=0 and therefore,


$$\begin{array}{r} e^{w\cdot Dz}=e^{-w\cdot Dz} \end{array}$$

for all $z\in Z_{D}\backslash \{w\}$ . In total, this yields


$$\int_{S}e^{w\cdot Dy}P_{x}^{⊥}Dy\;d\mu (y)=\sum_{z\in Z_{D}}t(z)(e^{w\cdot Dz}-e^{-w\cdot Dz})P_{w}^{⊥}(Dz)=0,$$

for all $w\in Z_{D}$ and thus also for μ -almost all w∈S .∎

The above proof strategy works only for Dirac measures aligned with the eigenvectors of D . However, there exist other discrete measures that are stationary points, as the following example shows. For the sake of simplicity, we restrict ourselves to the two-dimensional case with a positive definite matrix D and a symmetric combination of four Dirac measures. We further assume that D is diagonal; the case of a general symmetric D can be treated similarly with a rotation argument.

**Lemma 4.8.** *Let* n=2 *,* φ∈[0,2π) *and* D *be diagonal and positive definite. A discrete measure:*


(4.6) $$\mu_{\varphi }=\frac{1}{\left|X_{\varphi }\right|}\sum_{x\in X_{\varphi }}\delta_{x},\;where\;X_{\varphi }=\{X(\varphi ),X(\pi -\varphi ),X(\pi +\varphi ),X(2\pi -\varphi )\},$$

*is a stationary point of* $E_{D}$ *if and only if either* φ∈{0,π/2,π} *or*


(4.7) $$\begin{array}{lrr} & \frac{\tanh(\lambda {,}_{1}\;\cos^{2}\;\varphi )}{\tanh(\lambda {,}_{2}\;\sin^{2}\;\varphi )}=\frac{\lambda {,}_{2}}{\lambda {,}_{1}}, & \end{array}$$

*where* $\lambda {,}_{1},\lambda {,}_{2}$ *denote the diagonal entries of* D . *For any choice of* $\lambda {,}_{1},\lambda {,}_{2}>0$ , *there exists exactly one* φ∈(0,π/2) *that fulfils the condition in* equation (4.7).

*Proof.* Without loss of generality, we prove the statement for φ∈[0,π/2] *,* since otherwise it holds that (ψmod2π)∈[0,π/2] for a ψ∈{π−φ,π+φ,2π−φ} *,* and thus $\mu_{\varphi }=\mu_{\psi }$ .

It follows directly from lemma 4.6 that $\mu_{\varphi }$ is a stationary point if φ∈{0,π/2} . Therefore, it remains to show that $\mu_{\varphi }$ is a stationary point if and only if equation (4.7) is fulfilled. This means that we have to see when there exists a Lipschitz continuous V such that $\;dE_{D}(\mu_{\varphi },V)\neq 0$ .

We first fix x∈S and consider


(4.8) $$\begin{array}{rl} \int_{S}e^{x\cdot Dy}P_{x}^{⊥}Dy\;\;d\mu {\;}_{\varphi }(y)= & \frac{1}{4}((e^{x\cdot DX(\varphi )}-e^{-x\cdot DX(\varphi )})P_{x}^{⊥}DX(\varphi )\\ & +(e^{x\cdot DX(\pi -\varphi )}-e^{-x\cdot DX(\pi -\varphi )})P_{x}^{⊥}DX(\pi -\varphi )). \end{array}$$

Since n=2 , we can further write $P_{x}^{⟂}y=x^{⟂}\cdot y\;x{,}^{⟂}$ , where $x^{⟂}=(-x_{2},x_{1}{)}^{T}$ . We factor out $x^{⟂}$ to rewrite equation (4.8) as $E(x;\mu_{\varphi })\;x{,}^{⟂}$ with


$$\begin{array}{r} E(x;\mu_{\varphi })=(1/2)\left(\sinh(x\cdot DX(\varphi ))\;x{,}^{⟂}\cdot DX(\varphi )+\sinh(x\cdot DX(\pi -\varphi ))\;x{,}^{⟂}\cdot DX(\pi -\varphi )\right). \end{array}$$

Lemma 4.1 now gives us that


$$dE_{D}\left(\mu_{\varphi },V\right)=\sum_{x\in X_{\varphi }}E\left(x;\mu_{\varphi }\right)x^{⊥}\cdot V(x),$$

which can become zero for all admissible V if and only if $E(x;\mu_{\varphi })=0$ for all $x\in X_{\varphi }$ . Due to the symmetry properties of our measures $\mu_{\varphi }$ , it further holds that $E(x;\mu_{\varphi })$ is constant on $X_{\varphi }$ ; therefore, it suffices to consider x=X(φ) . Remembering that $X(\varphi )=(\cos\;\varphi ,\sin\;\varphi {)}^{T}$ , we derive


$$\begin{array}{rl} 2E(X(\varphi );\mu_{\varphi })= & \;\sinh(\lambda {\;}_{1}\;\cos{\;}^{2}\varphi +\lambda {\;}_{2}\;\sin{\;}^{2}\varphi )\;(-\lambda {\;}_{1}+\lambda {\;}_{2})\;\sin\varphi \cos\varphi \\ & +\sinh(-\lambda {\;}_{1}\;\cos{\;}^{2}\varphi +\lambda {\;}_{2}\;\sin{\;}^{2}\varphi )\;(\lambda {\;}_{1}+\lambda {\;}_{2})\;\sin\varphi \cos\varphi . \end{array}$$

Since φ∈(0,π/2) *,* the factor sin⁡φcos⁡φ cannot vanish, and the zeros of $E(X(\varphi );\mu_{\varphi })$ coincide with those of


sinh⁡(λ 1cos 2φ+λ 2sin 2φ)(−λ 1+λ 2)+sinh(−λ 1cos 2φ+λ 2sin 2φ)(λ 1+λ 2)(4.9)=sinh⁡(λ 1+(−λ 1+λ 2)sin 2φ)(−λ 1+λ 2)+sinh(−λ 1+(λ 1+λ 2)sin 2φ)(λ 1+λ 2).

This function obtains its minima at (φmod2π)∈{0,π} and its maxima at (φmod2π)∈{π/2,3π/2} and strictly increases or decreases, respectively, in between. Substituting these points into equation (4.9), we see that the minima are strictly negative and the maxima are strictly positive since $\lambda {,}_{1},\lambda {,}_{2}>0$ . Therefore, there exists exactly one zero in the interval (0,π/2) . Using the hyperbolic identity sinh⁡(x+y)=sinh⁡xcosh⁡y+cosh⁡xsinh⁡y in equation (4.9), we arrive at the criterion in equation (4.7).∎

**Remark 4.9.** *Importantly, the angle* φ *that fulfils* equation (4.7) *depends not only on the ratio of the eigenvalues of* D *but also on their magnitude since they appear separately within the hyperbolic tangent*.

Although the ratio of the eigenvalues does in general not determine the angle φ that fulfils equation (4.7), we can still make a qualitative prediction based on the ratio. The left-hand side of equation (4.7) decreases monotonically for φ∈[0,π/2) ; for $\lambda {,}_{1}=\lambda {,}_{2}$ , the condition is fulfilled for φ=π/4 . Therefore, the condition is fulfilled by some φ∈[0,π/4) if $\lambda {,}_{2}>\lambda {,}_{1}$ and by some φ∈(π/4,π/2] if $\lambda {,}_{1}>\lambda {,}_{2}$ . The numerical experiments in §5b show that the measures characterized by equation (4.7) are not only stationary points but also minimizers among empirical measures consisting of at most four Dirac measures. In the remainder of this section, we aim to characterize minimizers for positive definite matrices D in arbitrary dimensions n⩾2 .

### (b) Energy variation at the uniform distribution

To characterize minimizers for positive definite D , we start by identifying the cases when the uniform distribution is a stationary state. As we show in the following lemma, this can only be the case if the strength of the interaction does not depend on the direction, i.e. the eigenvalues of D all have the same absolute value.

**Lemma 4.10.** *The uniform distribution* $\mu =\frac{1}{\left|S^{n-1}\right|}H^{n}$ *is a stationary point of* $E_{D}$ *if and only if all eigenvalues* $(\lambda {,}_{i}{)}_{i=1}^{n}$ *of* D *have the same absolute value, i.e.* $|\lambda {,}_{i}|=\lambda$ *for some* λ∈ℝ .

*Proof.* To keep the notation simple, we treat here the case n=2 , leaving the general proof for n>2 to appendix C(a). Let us fix x∈S and determine φ∈[0,2π) such that $Dx/\|Dx\|=(\cos\;\varphi ,\sin\;\varphi {)}^{T}$ . Consider the integral


$$\begin{array}{rl} \int_{S}e^{x\cdot Dy}P_{x}^{⟂}Dy\;\;dH{,}^{2}(y) & =\int_{0}^{2\pi }e^{\left\|Dx\|\;\cos(\psi -\varphi \right)}P_{x}^{⟂}\left(D(\cos\;\psi ,\sin\;\psi {)}^{T}\right)\;\;d\psi =(*), \end{array}$$

which can be rewritten with a change of variables θ=ψ−φ as follows (recall that $P_{x}^{⟂}=\mathrm{Id}-xx^{T}$ ):


$$\begin{array}{rl} {}* & =\int_{0}^{2\pi }e^{\|Dx\|\cos\theta }\left(\cos\theta \left(D^{2}x/\|Dx\|-\|Dx\|x\right)+\sin\theta (Dx/\|Dx\|{)}^{⊥}\right)d\theta \\ & =\left(D^{2}x/\|Dx\|-\|Dx\|x\right)\underset{>0}{\underbrace{\int_{0}^{2\pi }e^{\|Dx\|\cos\theta }\cos\theta d\theta }}+(Dx/\|Dx\|{)}^{⊥}\underset{=0}{\underbrace{\int_{0}^{2\pi }e^{\|Dx\|\cos\theta }\sin\theta d\theta }}. \end{array}$$

From the above derivations, we see that (∗)=0 if and only if x is an eigenvector of $D^{2}$ . This holds true for μ -almost all x∈S if and only if $|\lambda {,}_{1}|=\left|\lambda {,}_{2}\right|$ . This automatically yields $\;dE_{D}(\mu ,V)=0$ if $|\lambda {,}_{1}|=\left|\lambda {,}_{2}\right|$ . It remains to show that this is also a necessary condition.

Without loss of generality, we assume that $|\lambda {,}_{1}|>\left|\lambda {,}_{2}\right|$ , where $\lambda {,}_{1}$ and $\lambda {,}_{2}$ are the eigenvalues corresponding to the eigenvectors $z_{1}$ and $z_{2}$ , respectively. Then, $\left(D^{2}x/\|Dx\|-\|Dx\|x\right)\cdot z_{2}$ is strictly negative on the set


$$\begin{array}{r} A=\{x\in S\;|\;(x\cdot z_{1})\in (|\lambda {,}_{2}/\lambda {,}_{1}|,1),\;(x\cdot z_{2})>0\}. \end{array}$$

Since μ(A)>0 we can find a Lipschitz continuous V such that $V\cdot z_{1}=0$ for μ -a.e. on S and


$$\begin{array}{r} V(x)\cdot z_{2}\left\{ \begin{array}{ll} >0 & \text{for a.e. }x\in A,\\ =0 & \text{for a.e. }x\in S\setminus A. \end{array}\right. \end{array}$$

For all such V it holds that $\;dE_{D}(\mu ,V)>0$ *,* which concludes the proof.∎

Since we already know that minimizers for D with at least one negative eigenvalue are Dirac measures, we can conclude that the uniform distribution is only a minimizer for D=Id⁡ .

**Corollary 4.11.** *The uniform distribution* $\mu =\frac{1}{\left|S^{n-1}\right|}H^{n}$ *minimizes* $E_{D}$ *if and only if* D=λId⁡ *for* λ⩾0 .

*Proof.* We only need to show that there are no other matrices D such that $E_{D}$ is minimized by μ ; the other direction has been treated in proposition 3.11. The measure μ can only be a minimizer if it is a stationary point. By lemma 4.10, this implies that all eigenvalues of D have to have the same absolute value. If such D has at least one negative eigenvalue, it is also the smallest eigenvalue. Thus, by theorem 3.1, the only minimizers are Dirac deltas placed at eigenvectors corresponding to the negative eigenvalue.∎

### (c) Perturbation of the identity

It is not clear whether an explicit computation of stationary points for an arbitrary positive definite matrix D with at least two distinct eigenvalues is possible, but some insight can be gained with asymptotic analysis. We consider the following perturbed energy:


$$\begin{array}{r} E{,}_{\varepsilon }(\mu ):=\int_{S}\int_{S}e^{x\cdot (\mathrm{Id}\;+\varepsilon M)y}\;\;d\mu (x)\;\;d\mu (y), \end{array}$$

where M is a diagonal matrix and |ε|≪1 is a small parameter. Using the second-order Taylor expansion of the exponential function, we can write


$$\begin{array}{lcr} & E_{\varepsilon }(\mu )\approx E_{D}(\mu )+\varepsilon \int_{S}\int_{S}e^{x\cdot y}x\cdot Myd\mu (x)d\mu (y)+\varepsilon^{2}\int_{S}e^{x\cdot y}(x\cdot My{)}^{2}d\mu (x)d\mu (y). & \text{(4.10)} \end{array}$$

For ε=0 we know that the unique minimizer $\mu_{0}$ is the uniform distribution on the sphere. Therefore, we use the following second-order asymptotic ansatz:


(4.11) $$\begin{array}{lcr} & \mu_{\varepsilon }:=\mu_{0}+\varepsilon \nu +\varepsilon^{2}w,\;\int_{S}\;d\nu =\int_{S}\;dw=0. & \end{array}$$

We stress that here we consider the energy as a function on the space of signed Radon measures on the sphere M(S) with the total variation norm and not on the space of probability measures P(S) with the Wasserstein metric as in §4a. For this reason, the perturbation here is a measure and not a vector field (cf. equation (4.1)).

Substituting equation (4.11) into equation (4.10) and neglecting higher-order terms, we derive


$$\begin{array}{r} E{,}_{\varepsilon }(\mu_{\varepsilon })-E{,}_{\varepsilon }(\mu_{0})\approx \;\varepsilon \;E_{D}(\mu_{0},\nu )+\varepsilon^{2}\;E_{D}(\mu_{0},w)+\varepsilon^{2}\;E_{D}(\nu )+2\varepsilon^{2}\int_{S}\int_{S}e^{x\cdot y}x\cdot My\;\;d\mu {,}_{0}(x)\;d\nu (y). \end{array}$$

Since further $y\mapsto \int_{S}e^{x\cdot y}\;d\mu_{0}(x)$ is constant on S , it follows that


$$\begin{array}{r} E_{D}(\mu_{0},\nu )=C(n)\int_{S}\;\;d\nu \;=\;0\;\text{and}\;E_{D}(\mu_{0},w)=C(n)\int_{S}\;\;dw\;=\;0. \end{array}$$

In particular, we see that the term $\varepsilon^{2}\omega$ from equation (4.11) does not contribute to the second-order expansion of the energy. Therefore, minimizing $E{,}_{\varepsilon }$ over all possible $\mu_{\varepsilon }$ satisfying equation (4.11) is equivalent to minimizing


$$\begin{array}{r} {\tilde{E}}_{\varepsilon }(\nu ):=\varepsilon^{2}\left(E_{D}(\nu )+2\int_{S}\int_{S}e^{x\cdot y}x\cdot My\;\;d\mu {,}_{0}(x)\;d\nu (y)\right) \end{array}$$

over all signed measures ν with ν(S)=0 . The first variation in the direction $\nu^{\prime }$ satisfying $\int_{S}\;\;d\nu {,}^{\prime }=0$ is given by


(4.12) $$\begin{array}{lrr} & \;d{\tilde{E}}_{\varepsilon }(\nu ,\nu^{\prime })=2\varepsilon^{2}\left(\int_{S}\int_{S}e^{x\cdot y}\;\;d\nu (x)\;\;d\nu {,}^{\prime }(y)+\int_{S}\int_{S}e^{x\cdot y}x\cdot My\;\;d\mu {,}_{0}(x)\;d\nu^{\prime }(y)\right). & \end{array}$$

Our goal is now to find an optimal measure ν , such that its first variation vanishes in any direction $\nu^{\prime }$ such that $\int_{S}\;\;d\nu {,}^{\prime }=0$ . To do so, we shall need the following two technical lemmas. To make the definition of the uniform distribution on the sphere rigorous, we denote by $H^{n}$ the n -dimensional Hausdorff measure and write $S^{n-1}$ instead of S .

**Lemma 4.12.** *Let* n⩾2 *and* $\mu_{0}=\frac{1}{\left|S^{n-1}\right|}H^{n}$ . *It holds that*


(4.13) $$\int_{S^{n-1}}e^{x\cdot y}x\;d\mu_{0}(x)=C_{1}y$$

*for any* $y\in S^{n-1}$ *, where the constant* $C_{1}$ *is positive and depends only on the dimension* n .

*Proof.* For the sake of simplicity, here we present the (more intuitive) proof for n=2 , leaving the general case n>2 to appendix C(b). We write $x=(\cos\;\varphi ,\sin\;\varphi {)}^{T}$ and $y=(\cos\;\psi ,\sin\;\psi {)}^{T}$ and derive that


$$\begin{array}{rl} 2\pi \int_{S}e^{x\cdot y}x\;\;d\mu {\;}_{0}(x) & =\int_{0}^{2\pi }e^{\cos(\varphi -\psi )}(\cos\varphi ,\sin\varphi {)}^{T}\;\;d\varphi \;=\int_{0}^{2\pi }e^{\cos\theta }(\cos(\psi +\theta ),\sin(\psi +\theta ){)}^{T}\;\;d\theta \\ & =(\cos\psi ,\sin\psi {)}^{T}\int_{0}^{2\pi }e^{\cos\theta }\cos\theta \;\;d\theta \;+\;(-\sin\psi ,\cos\psi {)}^{T}\int_{0}^{2\pi }e^{\cos\theta }\sin\theta \;\;d\theta , \end{array}$$

where we use the coordinate transform θ=φ−ψ and two trigonometric identities to separate the summands inside sine and cosine. Since $\int_{S}e^{\cos\theta }\sin\theta \;\;d\theta \;=\;0$ , this yields equation (4.13) with


$$C_{1}=\frac{1}{2\pi }\int_{0}^{2\pi }e^{\cos\theta }\cos\theta \;d\theta >0.$$

∎

**Lemma 4.13.** *Let* n⩾2 *and* $\mu_{0}=\frac{1}{\left|S^{n-1}\right|}H^{n}$ . *It holds that for any* $y\in S^{n-1}$


(4.14)∫Sn−1ex⋅yxi2 dμ0(x)=C2yi2+C3,1⩽i⩽n,

*where the constants* $C_{2}$ *and* $C_{3}$ *are positive and depend only on the dimension* n .

*Proof.* For the sake of simplicity, we again present the proof for n=2 ; the general case n>2 is treated in appendix C(c). Using the same arguments as in the previous proof, we derive


$$\begin{array}{rl} 2\pi \int_{S}e^{x\cdot y}x^{2}\;\;d\mu {\;}_{0}(x) & =\int_{0}^{2\pi }e^{\cos\theta }(\cos^{2}(\psi +\theta ),\sin^{2}(\psi +\theta ){)}^{T}\;\;d\theta \\ & =(\cos^{2}\psi ,\sin^{2}\psi {)}^{T}\int_{0}^{2\pi }e^{\cos\theta }\cos^{2}\theta \;\;d\theta +(\sin^{2}\psi ,\cos^{2}\psi {)}^{T}\int_{0}^{2\pi }e^{\cos\theta }\sin^{2}\theta \;\;d\theta , \end{array}$$

where the mixed terms containing cos⁡θsin⁡θ vanish due to symmetry. Further, since $\cos^{2}\;\psi +\sin^{2}\;\psi =1$ , we can write


$$\begin{array}{r} (\sin^{2}\;\psi ,\cos^{2}\;\psi {)}^{T}=(1,1{)}^{T}-(\cos^{2}\;\psi ,\sin^{2}\;\psi {)}^{T}. \end{array}$$

This yields equation (4.14) with positive constants:


$$\begin{array}{r} C_{2}=\frac{1}{2\pi }\int_{0}^{2\pi }e^{\cos\theta }(\cos^{2}(\theta )-\sin^{2}(\theta ))\;d\theta ,\;C_{3}=\frac{1}{2\pi }\int_{0}^{2\pi }e^{\cos\theta }\sin^{2}(\theta )\;d\theta . \end{array}$$

∎

Lemma 4.12 allows us to rewrite the second summand in equation (4.12) such that it contains y⋅My . Using lemma 4.13, we can then deduce that, up to constants, the measure $-\left(x\cdot Mx)\;\mu {,}_{0}(x\right)$ is a stationary point of ${\tilde{E}}_{\varepsilon }$ .

**Theorem 4.14.** *The measure*


$$\begin{array}{r} \;d\nu^{*}(x)=\left(\alpha \;x\cdot Mx+\beta \right)\;d\mu_{0}(x),\;\text{where }\alpha =-C_{1}/C_{2}\text{ and }\beta =-\int_{S}\alpha \;x\cdot Mx\;d\mu_{0}(x), \end{array}$$

*fulfils* $\int_{S}\;d\nu^{*}=0$ *and* $\;dE{,}_{\varepsilon }(\nu^{*},\nu^{\prime })=0$ *for all* $\nu^{\prime }$ *satisfying* $\;\int_{S}\;d\nu^{\prime }=0$ .

*Proof.* From the definition of β and $\int_{S}\;d\mu_{0}=1$ , it follows that $\int_{S}\;d\nu^{*}=0$ . With lemma 4.12, we write the optimality condition derived from equation (4.12) as


$$\begin{array}{r} \int_{S}\int_{S}e^{x\cdot y}\;\;d\nu (x)\;\;d\omega (y)=-C_{1}\int_{S}y\cdot My\;\;d\omega (y). \end{array}$$

Substituting $\nu^{*}$ into the left-hand side and using lemma 4.13, we get


$$\begin{array}{rl} \int_{S}\int_{S}e^{x\cdot y}\;d\nu^{*}(x)d\omega (y) & =\int_{S}\alpha \left(C_{2}y\cdot My+\mathrm{Tr}(M)C_{3}\right)d\omega (y)+\beta \int_{S}\int_{S}e^{x\cdot y}\;d\mu_{0}(x)d\omega (y)\\ & =\alpha C_{2}\int_{S}y\cdot My\;d\omega (y), \end{array}$$

where all terms that do not depend on y , including $\int_{S}e^{x\cdot y}\;\;d\mu {,}_{0}(x)$ , vanish due to $\int_{S}\;\;d\omega \;=\;0$ . Substituting $\alpha =-C_{1}/C_{2}$ completes the proof.∎

Theorem 4.14 gives us the following intuitive characterization. The measure $\mu_{\varepsilon }$ that optimizes the perturbed energy is obtained by taking mass from the uniform distribution where (x⋅Mx) is large and adding it where (x⋅Mx) is small. In other words, we expect minimizers of the energy $E_{D}$ with a positive definite matrix D to have more mass in regions that correspond to small eigenvalues of D than in regions that correspond to large ones. This intuition is in line with the results of the particle approximation in figure 3. Furthermore, in figure 5, we also observe that the density obtained in equation (4.11) with the measure $\nu^{*}$ from above can indeed be seen as a first-order approximation for small values of ε .

## 5. Numerical examples

To illustrate the obtained theoretical results, we perform a series of numerical experiments using a particle approximation of the energy from equation (1.2) with an ensemble of N particles $X=\left(X_{1},\ldots ,X_{N}\right)$ *,*


$$E_{D}\left(\mu_{N}(X)\right),\;\text{ where }\;\mu_{N}(X)=\frac{1}{N}\sum_{i=1}^{N}\delta_{X_{i}}.$$

We consider the following particle flow, introduced in [9],


$${\dot{X}}_{i}(t)=P_{X_{i}(t)}^{⊥}\left(\pm \frac{1}{J_{i}(X)}\sum_{j=1}^{N}e^{X_{i}(t)\cdot DX_{j}(t)}DX_{j}(t)\right),$$

with normalization factors $J_{i}(X)$ . If we choose the constant normalization


(5.1) $$\begin{array}{lrr} & J_{i}(X)=N, & \end{array}$$

this corresponds merely to a step-size rescaling of a standard gradient descent scheme for $E_{D}$ , which is called the (USA) flow in [9]. Choosing the normalization as the partition function


(5.2) $$\begin{array}{r} J_{i}(X)=\sum_{j=1}^{N}e^{X_{i}(t)\cdot DX_{j}(t)}, \end{array}$$

corresponds more closely to the self-attention dynamics and is labelled the SA flow in [9]. In what follows, we mostly use the normalization in equation (5.2), highlighting minor differences between the two formulations as appropriate. We use the explicit Euler discretization from equation (1.5) with step size τ>0 to obtain the following update:


(5.3) $$\begin{array}{r} X_{i}(t+\tau )=\Pi \left(X_{i}(t)\pm \frac{\tau }{J_{i}(X)}\sum_{j=1}^{N}e^{X_{i}(t)\cdot DX_{j}(t)}DX_{j}(t)\right). \end{array}$$

**Remark 5.1.** *For* N=1 *and this scheme reduces to the following power iteration in the limit* τ→∞ :


$$\begin{array}{r} X_{1}(t+\tau )=\Pi (DX_{1}(t)). \end{array}$$

*In this regard, the iteration in* equation (5.3) *can be seen as a method for approximating the largest eigenvalue and the corresponding eigenvector. We leave further analysis of this connection to future work*.

The source code for the experiments here is available at https://github.com/TimRoith/TransformerDynamics and uses Python [61], mainly building upon the packages NumPy [62], SciPy [63] and PyTorch [64].

### (a) Maximizers for positive definite matrices

**Figure 1.**
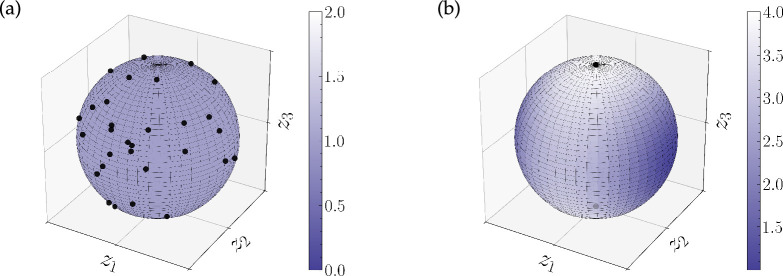
Discrete maximizers on the sphere for N=1 particles. The colour indicates the value of x⋅Dx at each point on the sphere. (a) For D=Id every single Dirac is a maximizer. We show the results for 30 different initializations (b) For D=diag(1,3,4) the final state is either (0, 0,1) or (0,0,−1).

To validate our results on maximizers, we first consider a simple set-up of a one-particle system, N=1 . We choose τ=0.075 and run the scheme in equation (5.3) for 1500 iterations. We only report the results for the adaptive normalization from equation (5.2), those for the constant normalization from equation (5.1) being essentially the same. For D=Id⁡ , we know that every single Dirac is a maximizer, which is indeed observed in figure 1a. Here, each random initialization on the sphere leads to a different final state. In fact, in this case, there is no evolution at all, and the particle stays at its initial position. If D is positive definite and has a strictly largest eigenvalue $\lambda {,}_{\text{max}}$ , theorem 3.1 shows that only Diracs at eigenvectors $z_{\text{max}}$ corresponding to $\lambda {,}_{\text{max}}$ are maximizers. This can be observed in figure 1b where the final state is either at $z_{\text{max}}$ or $-z_{\text{max}}$ .

**Figure 2.**
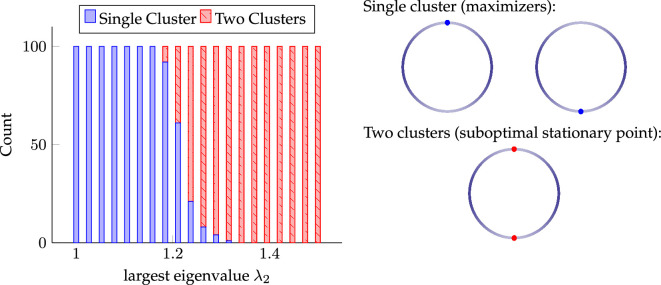
We study the trajectories for a symmetric positive definite matrix $D=\text{diag}(1,\lambda {,}_{2})$ with $\lambda {,}_{2}\in \left[1.,1.5\right]$ and 100 different initializations using 100 particles. We evaluate the number of clusters at the final iteration with the k -means implementation of the SciPy package [63]. The centre of each cluster is close to an eigenvector corresponding to an eigenvalue of maximal absolute value. For $\lambda {,}_{2}\approx 1$ , the evolution converges to the optimal state with a single cluster (blue, solid), while for bigger values, it tends to get stuck in the suboptimal stationary state with two clusters (red, hatched) from lemma 4.6.

For multiple particle systems with N>1 , lemma 4.6 suggests also that linear combinations of an eigenvector with its negative are stationary points. These linear combinations are not maximizers, but their basin of attraction depends on the eigenvalues of the matrix. In figure 2 (left), we plot the probability (i.e. the proportion of random initializations) of converging to a single cluster versus two clusters as function of the eigenvalues. We fix $\lambda {,}_{1}=1$ and vary $\lambda {,}_{2}$ between 1 and 1.5 . Note that, as discussed in lemma 4.8 and remark 4.9, the actual values of the eigenvalues matter; not just their ratio. For $\lambda {\;}_{2}\approx 1$ , the probability of converging to a single cluster is high, whereas for larger values $\lambda {,}_{2}≳1.4$ , most trajectories converge to two clusters. The results in figure 2 were obtained with the adaptive normalization from equation (5.2); however, we observed the same quantitative behaviour with the constant normalization from equation (5.1).

### (b) Minimizers for positive (semi-)definite matrices

**Figure 3.**
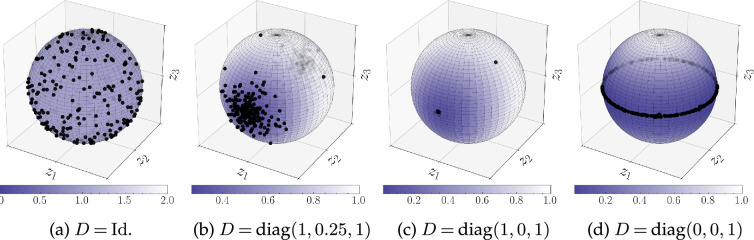
Final states for the minimization scheme after 10 000 steps with N=400 particles. The colour indicates the value of x⋅Dx at each point on the sphere. In (a), the uniform distribution is the minimizer of the energy. In (b), the particles do not form clusters at single Diracs but rather follow a smooth distribution on the sphere. In (c), any configuration with $(X_{i}{)}_{1}=(X_{i}{)}_{3}=0$ for all i is a minimizer. In (d), any configuration with $(X_{i}{)}_{3}=0$ for all i is a minimizer.

We now study discrete minimizers for positive definite matrices. In figure 3, we show how the matrix D influences the particle configuration to which the scheme in equation (5.3) converges. Here, too, we used the adaptive normalization from equation (5.2); the results for the constant one from equation (5.1) are largely the same.

**Figure 4.**
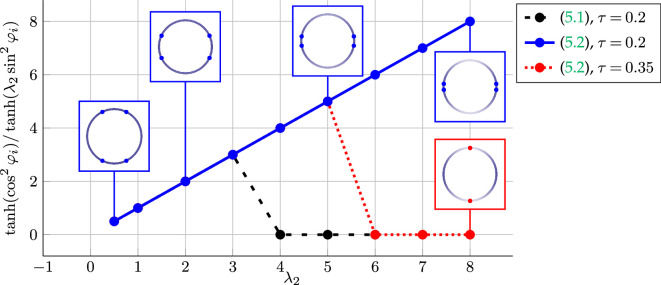
We consider minimizers for the matrix $D=\text{diag}(1,\lambda {,}_{2})$ . Starting with the initial configuration described in equation (5.4) , we compute the mean of $\tanh(\cos^{2}\;\varphi_{i})/\;\tanh(\lambda {,}_{2}\;\sin^{2}\;\varphi_{i})$ over all particles. For a small step size, the resulting curve is very close to the identity, as predicted by lemma 4.8. If $\lambda {,}_{2}\;\tau$ is too big, the dynamics converge to a suboptimal stationary point. We also compare the normalizations given by equations (5.1) and (5.2). We see that with the same step size τ=0.2 , the adaptive normalization in equation (5.1) yields faster convergence than the constant one in equation (5.2).


Furthermore, in figure 4, we illustrate the results of lemma 4.8 for matrices $D=\text{diag}(1,\lambda {,}_{2})$ with varying values $\lambda {,}_{2}\in \left[0.5,8\right]$ . We initialize N=4 particles as

(5.4)Xi=X(φi)withφi=(i−1)⋅π+π/4fori=1,…,4,

and let the scheme in equation (5.3) run for 10 000 iterations. From the final particle state, we compute the value $\tanh(\cos^{2}\;\varphi_{i})/\;\tanh(\lambda {,}_{2}\;\sin^{2}\;\varphi_{i})$ for each particle separately; lemma 4.8 tells us that this should be equal to $\lambda {,}_{2}$ for the minimizer. In figure 4, we observe that this holds true for the particle configurations computed with the discrete scheme. However, if the step size is too big compared to the value $\lambda {,}_{2}$ , the system instead converges to the two-cluster stationary point from figure 2. Here, we notice a slight difference between the two normalizations. The adaptive normalization from equation (5.2) allows choosing bigger step sizes compared to the constant normalization from equation (5.1), enabling faster convergence to the large-time limit.

We further investigate the validity of the asymptotic solution from theorem 4.14 in the two-dimensional case. Here, we deviate from the particle approximation and instead discretize the interval [−π,π) with N equidistant grid points $\Theta \in [-\pi ,\pi ]{,}^{N}$ and the associated points on the sphere $x_{1},\ldots ,x_{N}\in S^{1}$ . In this setting, we then aim to minimize


(5.5)E~ε(m)=∑i,j=1Nexi⋅(Id+εM)xjmi⋅mj,

where $m\in {\mathbb{R}}^{N}$ is a probability vector. Note that already, for n=3 , a more sophisticated quadrature rule would be required, e.g. the Lebedev quadrature on the sphere [65]. To deal with the simplex constraint for the vector m , we use exponentiated gradient descent, specifically mirror descent with the negative log-entropy as the distance generating function [66], which yields the update


$$\begin{array}{lcr} & m(\varepsilon {)}_{i}\leftarrow \frac{m_{i}e^{-\tau \nabla {\tilde{E}}_{\varepsilon }(m(\varepsilon ){)}_{i}}}{\sum_{j=1}^{N}m(\varepsilon {)}_{j}e^{-\tau \nabla \tilde{E}(m(\varepsilon ){)}_{j}}}=\mathrm{SoftMax}(\log(m(\varepsilon ))-\tau \nabla {\tilde{E}}_{\varepsilon }(m(\varepsilon ){)}_{i}. & \text{(5.6)} \end{array}$$

**Figure 5.**
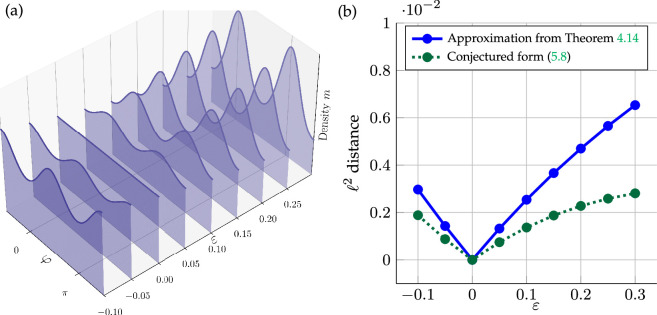
Numerical study of the asymptotic solution from theorem 4.14 in two dimensions. (a) The probability vectors m(ϵ) computed using equation (5.5) with 500 steps for τ=0.1. (b) The $l^{2}$ approximation error for the first-order expansion in equation (5.7) (blue, solid) and the conjectured form in equation (5.8) (green, dotted)

We take the perturbation matrix as M=diag(0,1) *,* that is, the perturbed matrix D is given by $D_{\varepsilon }=\text{diag}(1,1+\varepsilon ).$ Recall the asymptotic expansion in equation (4.10). As noted in §4c, the contribution of the term $\varepsilon^{2}\omega$ vanishes in the second-order expansion of the energy, and we are left with a solution:


(5.7) $$\begin{array}{lcr} & \mu_{\varepsilon }^{*}=\mu_{0}+\varepsilon \nu^{*}, & \end{array}$$

where $\nu^{*}$ is as in theorem 4.14. We note that this measure has a Lebesgue density that can be evaluated at the grid points in Θ ; we denote the resulting vector by $\;d\mu_{\varepsilon }^{*}{|}_{\Theta }$ . In figure 5, we compare this solution to the vector m(ε) obtained by solving equations (5.5)–(5.6). The vector m(ε) for different values of ε is shown in figures 5a and 5b, we plot the $\ell^{2}$ error ${\left|m(\epsilon )-d\mu_{\varepsilon }^{*}{|}_{\Theta }\right|}_{2}$ .

Beyond the first-order expansion in equation (5.7), we conjecture that m(ε) behaves as follows:


(5.8) $$\begin{array}{lrr} & \;d\mu_{\varepsilon }^{\text{guess}}(\theta )∼\exp(\Upsilon (\varepsilon )\;\cos(2\theta )), & \end{array}$$

where Υ(ε) is a function to be determined. Taking a second-order Taylor expansion Υ(ε) , we estimate the coefficients via linear regression with the given vectors m(ε) as data points and obtain $\Upsilon (\varepsilon )\approx 1/5\;\varepsilon {,}^{2}+e/2\;\varepsilon$ . The $\ell^{2}$ error of this approximation is shown in figure 5b and is lower than that of the first-order expansion in equation (5.7). We leave the analysis of this ansatz to future work.

### (c) Maximizers for negative definite and indefinite matrices

**Figure 6.**
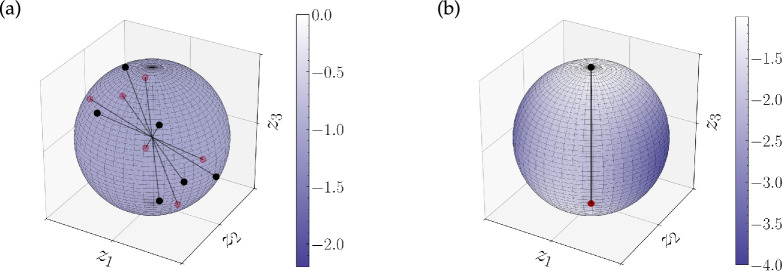
Discrete maximizers on the sphere for negative definite matrices obtained with N=100 particles. We visualize the two-cluster final states by connecting the two components of each cluster corresponding to the same run with a line, assigning different colours to the two opposite clusters. The colour of the sphere indicates the value of x⋅Dx at each point on the sphere. (a) For *D* = −Id a single final state has clusters at both z and −z for any z∈S . For clarity, we only show results for 6 different initializations. (b) For *D* = −diag(1,3,4) a single final state has clusters both at (0,0,1) and (0,0,−1). We show the results for 100 different initializations.

We proceed to numerical examples for §3d, i.e. maximization of the energy corresponding to a negative definite matrix. We take a system of N=100 particles and consider the two matrices from figure 1 multiplied by −1 . The results are shown in figure 6. We observe that a single final state consists of clusters at ±z , where z is an eigenvector corresponding to the smallest eigenvalue, in agreement with theorem 3.17. As shown there, the behaviour does not change if one of the eigenvalues is zero, as only the eigenvectors corresponding to the smallest eigenvalue are relevant. For this reason, we do not consider the semi-definite case separately. The results here are not affected by the choice of the normalization; we only show the ones obtained with that in equation (5.2).

**Figure 7.**
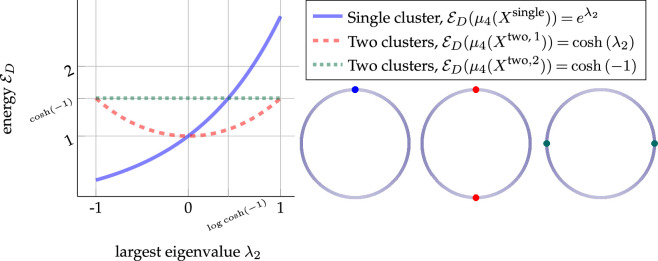
Energies of the states $X^{\text{single}}=\left((0,1)\right)$ in blue, $X^{\text{two},1}=\left((0,1),(0,-1)\right)$ in red and $X^{\text{two},2}=\left((1,0),(-1,0)\right)$ in green for the matrix $D=\text{diag}(-1,\lambda {,}_{2})$ with varying values of $\lambda {,}_{2}$ .

Finally, we turn to the case of indefinite matrices. As noted in remark 3.5, for a matrix D that is not negative definite, a Dirac delta placed at the eigenvector corresponding to the largest eigenvalue may not be a maximizer. This can be observed numerically as shown in figure 7 where we plot the energies of one- and two-cluster states for $D=\text{diag}(-1,\lambda {,}_{2})$ with $\lambda {,}_{2}\in \left[-1,1\right]$ .

## 6. Conclusion

In this work, we studied a mathematical model of self-attention layers used in the transformer architecture. Building upon [9], we analysed a continuum limit in the space of probability measures on a sphere. To understand the underlying geometry, we studied a new optimal transport distance $W_{m,2}$ with non-local mobility. We proved that the space of probability measures with this distance is a geodesic space and characterized absolutely continuous curves in this space. This allowed us to interpret the continuity equation (2.5) as curves of maximal slope of the interaction energy and to analyse the large-time behaviour using the energy dissipation property, showing that the dynamics converge to a stationary point of the interaction energy.

We analysed these critical points (in particular, minimizers and maximizers) for various types of interactions determined by the matrix D in equation (1.2). These results are listed in table 1. We find that the positions of stationary points are strongly connected to normalized eigenvectors of D , which form a strict subset of S in the case D≠λId⁡ . In other words, the regions where clusters appear do not only depend on the initial configuration, but also on the interaction matrix itself. This could be related to mode collapse often observed in practice. It is an interesting question to understand whether an alternative, rotation-invariant architecture could prevent mode collapse.

**Table 1.** Summary of results on minimizers/maximizers of the interaction energy in equation (1.2). We denote by $z_{\min}$ and $z_{\max}$ the eigenvectors that correspond to the smallest, respectively largest, eigenvalue of D .

property of D	minimizers	maximizers
top rule positive definite	symmetric w.r.t. all eigenvectors (corollary 3.10 and §5b)	$\mu =\delta_{z_{\max}}$ (theorem 3.1 and §5a)
mid-rule positive semi-definite	any μ concentrated on N(D) (theorem 3.4 and §5b)	$\mu =\delta_{z_{\max}}$ (theorem 3.1 )
negative (semi-)definite	$\mu =\delta_{z_{\min}}$ (theorem 3.1)	$\mu =1/2\;(\delta_{z_{\min}}+\delta_{-z_{\min}})$ (corollary 3.16 and §5c)
indefinite	$\mu =\delta_{z_{\min}}$ (theorem 3.4)	$\left|\lambda_{\max}\right|$ maximal: $\mu =\delta_{z_{\max}}$ (theorem 3.1 and §5c)

Several further questions remain open for future work: as already discussed, it would be interesting to study the optimal transport distance for mobilities $m_{\mu }$ that cannot be bounded from below, which is the case, for example, in problems of opinion dynamics where the Gaussian kernel on the Euclidean space is often used. In this case, the metric $W_{m,2}$ is no longer equivalent to $W_{2}$ . So far, we have only shown that equation (2.6) represents gradient flows in $\left(P(M),W_{m,2}\right)$ using the concept of curves of maximal slope. We do not know if these curves satisfy the slightly stronger energy variational inequality, which would yield an easy stability estimate for solutions of equation (2.6).

From a practical point of view, an even more interesting direction is studying more general flows in $W_{m,2}$ that correspond to non-symmetrical matrices D in equation (1.2), which is common in transformer architectures. As mentioned above, basic properties of the distance carry over to the non-symmetric case, but characterizing the stationary states is non-trivial; one possibility is splitting the effective velocity fields into a dissipative and a (generalized) divergence-free part, similar to non-symmetric Fokker–Planck equations.

Finally, to justify the use of the continuum limit for studying the practical behaviour of transformers, one needs to establish convergence of discrete time-stepping in arbitrary time intervals. Moreover, it is worth studying how the step size influences the behaviour of the system and what effect weight-sharing would have.

## Data Availability

This article has no additional data.

## Appendix A. Proofs of Section 2

### A.1. Continuity equation on manifolds

Let M be a compact, n -dimensional Riemannian manifold and $TM={⊔}_{x\in M}T_{x}M$ its tangent bundle. Although TM is not a vector space, the tangent bundle TM itself can be considered as 2n -dimensional Riemannian manifold. For its proper definition and the topology on TM , we refer to [67, ch. 3 (The Tangent Bundle)]. Velocity fields on manifolds are maps V:M→TM such that $\pi \circ V=Id_{M}$ , where π:TM→M is the projection map sending each vector in $T_{x}M$ to x . We shall regularly commit the mild crime of interpreting V(x) as an element in $T_{x}M$ instead of TM . Let I=(0,T) be an open interval, ${\left(\mu_{t}\right)}_{t\in I}$ be a Borel family of probability measures on M and $v:(x,t)\in M\times I\mapsto v_{t}(x)\in T_{x}M$ be a time-dependent Borel velocity field such that

(A 1)
$$\begin{array}{lrr} & \int \int |v_{t}(x)|\;d\mu_{t}\;dt<\infty , & \end{array}$$

where $|\cdot |:TM_{x}\to \left[0,+\infty \right)$ denotes the norm induced by the inner product of the Riemannian structure. The continuity equation holds in the sense of distributions if

(A 2)
$$\begin{array}{lrr} & \int_{\left(0,T\right)}\int_{M}\partial_{t}\varphi (x,t)+\langle D\varphi (x,t),v_{t}(x)\rangle \;d\mu_{t}\;dt=0\;\forall \varphi \in C_{c}^{1}(M\times (0,T)). & \end{array}$$

Here, Dφ denotes the differential of the map x∈M↦φ(t,x) for a fixed t∈[0,T] .

**Proposition A.1** (Properties). *Solutions to the continuity equation have the following properties:*

— *Continuous representative: Let* $\mu_{t}$ *be a Borel family of probability measures satisfying* *equation (A 2)* *for a Borel vector field* $v_{t}$ *satisfying* *equation (A 1)*. *Then there exists a narrowly continuous curve* $t\in [0,T]\to {\tilde{\mu }}_{t}\in P(M)$ *such that* $\mu_{t}={\tilde{\mu }}_{t}$ *for a.e*. t∈(0,T) . *Moreover, if* $\varphi \in C_{c}^{1}(M\times [0,T])$ *and* s⩽r∈[0,T] *we have* [53, Lemma 8.1.2]:
  (A3)
  $$\begin{array}{lrr} & \int_{M}\varphi (x,r)\;d{\tilde{\mu }}_{r}-\int_{M}\varphi (x,s)\;d{\tilde{\mu }}_{s}=\int_{s}^{r}\int_{M}\partial_{t}\varphi +D\varphi (v_{t})\;d\mu_{t}\;dt. & \end{array}$$
— *Time rescaling: Let* $t:s\in [0,T^{\prime }]\;\to \;t(s)\in [0,T]$ *be a strictly increasing absolutely continuous map with absolutely continuous inverse* $s≔t^{-1}$ . *Then* $\left(\mu_{t},v_{t}\right)$ *is a distributional solution of the continuity equation if and only if* [68, Lemma 8.1.3]
  $\begin{array}{r} \hat{\mu }:=\mu \;\circ \;t,\hat{v}\;=t^{\prime }\;v\;\circ \;t\; \end{array}$ is a distributional solution of the continuity equation on $\left(0,T^{\prime }\right)$ .
— *Gluing solutions: Let* $\{\mu_{t}{\}}_{t\in [0,T_{1}]},\{\nu_{t}{\}}_{t\in [0,T_{2}]}$ *be two narrowly continuous curves in* P(M) *with* $\mu_{T_{1}}=\nu_{0}$ . *Let further* $\{v{\}}_{t\in [0,T_{1}]},\{w{\}}_{t\in [0,T_{2}]}$ *be the corresponding Borel velocity fields such that* *equation (A 3)* *is satisfied. Then* $\{\eta_{t}{\}}_{t\in [0,T_{1}+T_{2}]}$ *and* $\{u_{t}{\}}_{t\in [0,T_{1}+T_{2}]}$ *defined by*
  $$\begin{array}{r} \eta_{t}:=\left\{ \begin{array}{ll} \mu_{t} & \text{if }t\in [0,T_{1}],\\ \nu_{t-T_{1}} & \text{if }t\in (T_{1},T_{1}+T_{2}], \end{array}\right.\;u_{t}:=\left\{ \begin{array}{ll} v_{t} & \text{if }t\in [0,T_{1}],\\ w_{t-T_{1}} & \text{if }t\in (T_{1},T_{1}+T_{2}], \end{array}\right. \end{array}$$
  *satisfy* *equation (A 3)* *[* 69 *, Lemma 4.4]*.

### A.2. Proof of Theorem 2.2

We follow the proof strategy from [69] for the ‘flat’ Euclidean case, but since TM is not a vector space, modifications are required. We start by establishing a compactness result for solutions of continuity equations with finite energy. For our purposes, we define the ‘lifted’ flux $J_{t}\in P(TM\times M)$ in duality with $C_{c}(TM\times M)$ (see [70, Theorem 7.2]) by

(A 4)
$$\begin{array}{lrr} & \int_{TM\times M}\varphi (w,y)\;dJ_{t}(w,y)=\int_{M}\int_{M}\varphi (v_{t}(x),y)\;d\mu_{t}(x)\;d\mu_{t}(y)\;\forall \varphi \in C_{c}(TM\times M). & \end{array}$$

Notably, $\left(\mu_{t},J_{t}\right)$ solve the continuity equation in the sense that for all s⩽r∈[0,T] :

(A 5)
$$\begin{array}{lrr} & \int_{M}\varphi_{r}\;d\mu_{r}-\int_{M}\varphi_{s}\;d\mu_{s}=\int_{s}^{r}\int_{M}\partial_{t}\varphi \;d\mu_{t}\;dt+\int_{s}^{r}\int_{TM\times M}\tilde{D\varphi }\;dJ_{t}\;dt\;\forall \varphi \in C^{1}(M\times [0,T]), & \end{array}$$

where $\tilde{D\varphi }:(w,y)\mapsto \left\langle D\varphi (\pi (w)),w\right\rangle$ is the extension of Dφ on to TM×M that is constant along y∈M . Further, we define J∈P(TM×M×[0,T]) in duality with $C_{c}(TM\times M\times [0,T])$ by

$$\int_{TM\times M\times (0,T)}\varphi dJ=\int_{0}^{T}\int_{TM\times M}\varphi dJ_{t}dt\;\forall \varphi \in C_{c}(TM\times M\times [0,T]).$$

**Lemma A.2.** *Let* $\left(\mu^{n},v^{n}\right)$ *be a sequence in* CE(0,T) *with*

$$\begin{array}{r} \underset{n}{\sup}\left\{\int_{0}^{1}\int_{M}m_{\mu }(x)|v_{t}^{n}(x){|}^{2}\;d\mu_{t}^{n}(x)\;dt\right\}<+\infty . \end{array}$$

*Then there exists a subsequence and a couple* (μ,J) *satisfying the continuity equation in the sense of* *equation (A 5)* *such that*

$$\begin{array}{r} \mu_{t}^{n}⇀\mu_{t}\;\forall t\in [0,T]\;\text{and}\;J^{n}⇀J, \end{array}$$

*and for the map* g:(v,p)∈TM×M↦(π(v),p) *one has*

(A 6)
$$\begin{array}{lrr} & g_{\#}J_{t}=\mu_{t}⊗\mu_{t}\;\text{for a.e. }t\in (0,T). & \end{array}$$

*Proof.* **Step 1** (Convergence of J ):

The estimate

$$\begin{array}{r} \underset{n}{\sup}\;\int_{0}^{T}\int_{TM\times M}|w{|}^{2}\;dJ_{t}^{n}(w,x)\;dt⩽\frac{1}{C}\underset{n}{\sup}\;\int_{0}^{T}\int_{TM}m_{\mu_{t}^{n}}|v_{t}^{n}{|}^{2}\;d\mu_{t}^{n}\;dt<\infty \end{array}$$

combined with the fact that M is compact and [53, Remark 5.1.5] implies tightness of J∈P(TM×M×[0,T]) . By disintegrating J , we obtain a Borel family $J_{t}$ such that $\;dJ=dJ_{t}\;dt$ . Since M is compact $\mu_{0}^{n}$ is tight, and we extract a further subsequence such that $\mu_{0}^{n}\;⇀\;\mu_{0}$ .

**Step 2** (Convergence of $\mu_{t}$ ):

Consider a function $\varphi \in C^{1}(M)$ and for t∈[0,T] set $\zeta :(v,y,t)\in TM\times M\times [0,T]\;\mapsto \;\chi_{\left[0,t\right]}\langle D\varphi (\pi (v)),v\rangle$ . Since the discontinuity set of ζ is concentrated on N=TM×M×{0,t} and |F|(N)=0 , general convergence theorems (see, e.g. [3, Prop. 5.1.10]) imply

limn→∞∫0t∫TM×MD~φ dJtn dt=limn→∞∫TM×M×[0,T]ζdJn(A 7)=∫TM×M×[0,T]ζdJ=∫0t∫TM×MD~φ dJt dt.

Let us fix a t∈(0,T] . Since M is compact, $\mu_{t}^{n}$ is tight, and we can extract from any subsequence a further subsequence such that $\mu_{t}^{n}$ converges narrowly. Then by equations (A 5) and (A 7) and the fact that $C^{1}$ is dense in $C^{0}$ , we know that all subsequences have the same limit. Therefore, $\mu_{t}^{n}⇀\mu_{t}\in P(M)$ for a particular $\mu_{t}$ . By the previous calculations, we also immediately obtain that (μ,J) satisfy the continuity equation in the sense of equation (A 5). To show equation (A 6), we observe that since M is compact

$$\begin{array}{r} g_{\#}J_{t}^{n}=\mu_{t}^{n}⊗\mu_{t}^{n}⇀\mu_{t}⊗\mu_{t}\;\forall t\in [0,T]. \end{array}$$

∎

*Proof of Theorem 2.2.* **Step 1**:

Let $(\mu^{n},v^{n})\in CE(0,1)$ be a minimizing sequence of the functional in equation (2.3) for some $\mu_{0},\mu_{1}$ . Then the conditions of lemma A.2 are met, and we obtain

$$\begin{array}{r} \mu_{t}^{n}⇀\mu_{t}\in P(M)\;\forall [0,T]\;\text{and}\;J^{n}⇀J\in P(TM\times M\times [0,T]), \end{array}$$

where the limit satisfies the continuity equation in the sense of equation (A 5). Equation (A 6) in particular implies that J can be disintegrated in the following way:

$$dJ=du_{t,x}(v)d\mu_{t}(x)d\mu_{t}(y)dt,$$

where $u_{t,x}(v)\in P(TM_{p}=\pi^{-1}(x))$ . Using [53, Lemma 5.1.7], we now show that for ${\bar{u}}_{x,t}=Mean(u_{t,x})=\int_{TM_{x}}v\;du_{t,x}(v)$ it holds that

$$\begin{array}{rl} W_{m,2}(\mu_{0},\mu_{1}{)}^{2}= & \lim_{n\to \infty }\int_{0}^{1}\int_{M}m_{\mu_{t}^{n}}(x)|v_{t}^{n}(x){|}^{2}\;d\mu_{t}^{n}(x)\;dt\\ = & \lim_{n\to \infty }\int_{0}^{1}\int_{TM\times M}K(\pi (v),y)|v{|}^{2}\;dJ_{t}^{n}(v,y)\;dt=\int_{0}^{1}\int_{TM\times M}K(\pi (v),y)|v{|}^{2}\;dJ_{t}(v,y)\;dt\\ = & \int_{0}^{1}\int_{M}\int_{M}K(x,y)\;d\mu_{t}(y)\int_{TM_{p}}|v{|}^{2}\;du_{x,t}(v)\;d\mu_{t}(x)\;dt\\ ⩾ & \int_{0}^{1}\int_{M}m_{\mu_{t}}(x)\int_{TM_{x}}|v{|}^{2}\;d\delta_{{\bar{u}}_{x,t}}(v)\;d\mu_{t}(x)\;dt=\int_{0}^{1}\int_{M}m_{\mu_{t}}(x)|{\bar{u}}_{x,t}{|}^{2}\;d\mu_{t}(x)\;dt, \end{array}$$

where in the last line, we used Jensen’s inequality. Since $D\varphi (x):TM_{x}\to \mathbb{R}$ is linear and (μ,J) satisfy equation (A 5), this implies that $\left(\mu ,v=({\bar{u}}_{x,t}{)}_{t\in [0,T]}\right)\in CE(0,1)$ and for this couple the infimum in equation (2.3) is obtained.

**Step 2**:

Proposition A.1 and a linear time rescaling show that

(A 8)
$$\begin{array}{lrr} & W_{m,2}^{2}(\mu_{0},\mu_{T})=\inf\left\{T\int_{0}^{T}\int_{M}m_{\mu_{t}}|v_{t}{|}^{2}\;d\mu_{t}\;dt:\;(\mu_{t},v_{t})\in CE(0,T;\mu_{0}\to \mu_{T})\right\}. & \end{array}$$

We denote by ${\bar{W}}_{m,2}(\mu ,\nu )$ the infimum in equation (2.4) and show that indeed ${\bar{W}}_{m,2}(\mu ,\nu )=W_{m,2}(\mu ,\nu )$ . By Hölder’s inequality, we immediately obtain that ${\bar{W}}_{m,2}(\mu ,\nu )⩽W_{m,2}(\mu ,\nu )$ . To show the reverse, we follow the arguments of [69, Theorem 5.4] and define for (μ,v)∈CE(0,T;μ→ν) :

$$\begin{array}{r} s_{\epsilon }(t)≔\int_{0}^{t}{\left(\epsilon +\int_{M}m_{\mu_{t}}|v_{t}{|}^{2}\;d\mu_{t}\right)}^{1/2}\;dr\;\text{for }t\in [0,T]. \end{array}$$

Then $s_{\epsilon }$ is strictly increasing, $s_{\epsilon }^{\prime }⩾\epsilon$ and $s_{\epsilon }(0,T)=\left(0,S_{\epsilon }\right)$ with $S_{\epsilon }≔s_{\epsilon }(T)$ , so that its inverse map $t_{\epsilon }:[0,S_{\epsilon }]\to \left[0,T\right]$ is well defined and Lipschitz continuous and

$$\begin{array}{r} t_{\epsilon }^{\prime }\;\circ \;s_{\epsilon }:={\left(\epsilon +\int_{M}m_{\mu_{t}}|v_{t}{|}^{2}\;d\mu_{t}\right)}^{-1/2}\;\text{for a.e. }t\in (0,T). \end{array}$$

By proposition A.1, we have that for $\mu^{\epsilon }:=\mu \;\circ \;t_{\epsilon }$ , $v^{\epsilon }:=t_{\epsilon }^{\prime }\;v\;\circ \;t_{\epsilon }$ the couple $(\mu^{\epsilon },v^{\epsilon })\in CE(0,S_{\epsilon };\mu ,\nu )$ and

$$\begin{array}{rl} W_{m,2}^{2}(\mu ,\nu ) & ⩽S_{\epsilon }\int_{0}^{S_{\epsilon }}\int_{M}m_{\mu_{t}^{\epsilon }}|v_{t}^{\epsilon }|\;d\mu_{t}^{\epsilon }\;ds\\ & =S_{\epsilon }\int_{0}^{T}\frac{\int_{M}m_{\mu_{t}}|v_{t}{|}^{2}\;d\mu_{t}}{\epsilon +\int_{M}m_{\mu_{t}}|v_{t}{|}^{2}\;d\mu_{t}}{\left(\epsilon +\int_{M}m_{\mu_{t}}|v_{t}{|}^{2}\;d\mu_{t}\right)}^{1/2}\;dt, \end{array}$$

with the last term being smaller or equal to $S_{\epsilon }^{2}$ . Sending ϵ→0 , we obtain

$$\begin{array}{r} W_{m,2}(\mu ,\nu )=\int_{0}^{T}{\left(\int_{M}m_{\mu_{t}}|v_{t}{|}^{2}\;d\mu_{t}\right)}^{1/2}\;dt\;\text{for all }(\mu ,v)\in CE(0,T;\mu \to \nu ) \end{array}$$

and hence, $W_{m,2}(\mu ,\nu )={\bar{W}}_{m,2}(\mu ,\nu )$ . This in particular implies that for every minimizer (μ,v)∈CE(0,1;μ→ν) of the functional in equation (2.3), the equality

$$\begin{array}{r} {\left(\int_{0}^{1}\int_{M}m_{\mu_{t}}|v_{t}{|}^{2}\;d\mu_{t}\;dt\right)}^{1/2}=\int_{0}^{1}{\left(\int_{M}m_{\mu_{t}}|v_{t}{|}^{2}\;d\mu_{t}\right)}^{1/2}\;dt \end{array}$$

holds, which is only the case when $\int_{M}m_{\mu_{t}}|v_{t}{|}^{2}\;d\mu_{t}$ is constant for a.e. t∈(0,T) , implying by a further time rescaling argument∎

(A 9)
$$W_{m,2}(\mu_{s},\mu_{t})=|s-t|\;W_{m,2}(\mu_{0},\mu_{1})\;\forall \;0⩽s⩽t⩽1.$$

### A.3. Proof of Lemma 2.4

*Proof of Lemma 2.4.* If (μ,v)∈CE(0,T) and $\int_{0}^{T}{\left(\int_{M}m_{\mu_{t}}|v_{t}{|}^{2}\;d\mu_{t}\right)}^{1/2}\;dt<+\infty$ then by equation (2.4), we have

$$\begin{array}{r} W_{m}(\mu_{s},\nu_{r})⩽\int_{s}^{r}{\left(\int_{M}m_{\mu_{t}}|v_{t}{|}^{2}\;d\mu_{t}\right)}^{1/2}\;dt\;\forall 0⩽s⩽r⩽T. \end{array}$$

On the other hand, if $\mu_{t}$ is an absolutely continuous curve, then by a standard reparametrization argument [53, Lemma 1.1.4], we may assume $\mu_{t}$ to be Lipschitz. For N∈ℕ , we set the step size as $\tau =T2^{-N}$ and choose a family of constant-speed geodesics $(\mu^{k,N},v^{k,N})\in CE((k-1)\tau ,k\tau ;\mu_{(k-1)\tau }\to \mu_{k\tau })$ *,* k∈{1,...,N} such that for t∈((k−1)τ,kτ)

$$\tau \int_{M}m_{\mu_{t}}{\left|v_{t}\right|}^{2}\;d\mu_{t}\overset{\left(A8\right)}{=}\frac{1}{\tau }W_{m}^{2}\left(\mu_{(k-1)\tau },\mu_{k\tau }\right)⩽\frac{1}{\tau }{\left(\int_{(k-1)\tau }^{k\tau }|\dot{\mu }|(t)dt\right)}^{2}\overset{\text{ Hölder }}{⩽}\int_{(k-1)\tau }^{k\tau }|\dot{\mu }|(t{)}^{2}\;dt.$$

Gluing all geodesics together by proposition A.1, we obtain a curve $(\mu^{N},v^{N})\in CE(0,1)$ . Lemma A.2 gives us a subsequence, still denoted by N , and a couple $(\tilde{\mu },\tilde{v})\in CE(0,1)$ such that $\mu_{t}^{N}\;⇀\;{\tilde{\mu }}_{t}$ and $J\;⇀\;\tilde{J}$ . By construction, ${\tilde{\mu }}_{t}$ and $\mu_{t}$ coincide on the dense (in [0,T] ) set $\{0\}\cup \left\{\frac{T}{M}2^{-N}:M,N\in \mathbb{R},M⩽N\right\}$ . Since both ${\tilde{\mu }}_{t}$ and $\mu_{t}$ are narrowly continuous ${\tilde{\mu }}_{t}=\mu_{t}$ must hold. Again, equation (A 6) implies that J can be disintegrated in the following way:

$$dJ=du_{t,x}(v)d\mu_{t}(x)d\mu_{t}(y)dt,$$

where $u_{t,x}(v)\in P(TM_{x}=\pi^{-1}(x))$ . Then $(\mu ,\tilde{v})\in CE(0,T)$ with ${\tilde{v}}_{t}≔\int_{TM_{x}}w\;du_{t,x}(w)$ and

(A 10)
$$\begin{array}{r} \begin{array}{rl} \int_{0}^{T}\int_{M}m_{\mu_{t}}|{\tilde{v}}_{t}{|}^{2}\;d\mu_{t}\;dt & \overset{\text{Jensen}}{⩽}\int_{0}^{T}\int_{M}\int_{M}K(x,y)\;d\mu_{t}(y)\int_{TM_{p}}|v{|}^{2}\;du_{x,t}(v)\;d\mu_{t}(x)\;dt\\ & \;⩽\int_{0}^{T}\int_{TM\times M}K(\pi (v),y)|v{|}^{2}\;dJ_{t}(v,y)\;dt\\ & \;⩽\underset{n\to \infty }{lim inf}\int_{0}^{T}\int_{TM\times M}K(\pi (v),y)|v{|}^{2}\;dJ_{t}^{n}(v,y)\;dt\\ & =\underset{n\to \infty }{lim inf}\int_{0}^{T}\int_{M}m_{\mu_{t}^{n}}|v_{t}^{n}{|}^{2}\;d\mu_{t}\;dt⩽\int_{0}^{T}|\dot{\mu }{|}^{2}(t)\;dt. \end{array} \end{array}$$

Since $(\mu ,\tilde{v})\in CE(0,T)$ , we have that

$$|\dot{\mu }|(t)⩽{\left(\int_{M}m_{\mu_{t}}|v_{t}{|}^{2}\;d\mu_{t}\right)}^{1/2}\;\text{for a.e. }t\in (0,T).$$

Finally, for equation (A 10) to hold $|\dot{\mu }|(t)={\left(\int_{M}m_{\mu_{t}}|v_{t}{|}^{2}\;d\mu_{t}\right)}^{1/2}$ must hold for a.e. t∈(0,T) ∎

### A.4. Proof of Lemma 2.6

*Proof of Lemma 2.6.* From theorem 2.3, we know that the distances $W_{2}$ and $W_{m,2}$ are equivalent. Therefore, we can assume absolute continuity with respect to $W_{2}$ . Further, by a standard rescaling argument (e.g. [53, Lemma 1.1.4] or [53, Lemma 8.1.3]), it is enough to prove equation (2.7) for 1 -Lipschitz curves (w.r.t. $W_{2}$ ), i.e. we only need to consider absolutely continuous curves $(\mu_{t},v_{t})\in CE(0,1;\mu \to \nu )$ such that

$$\begin{array}{r} \int_{M}|v_{t}(x){|}^{2}\;d\mu_{t}(x)=1\;\text{for a.e. }t\in (0,T). \end{array}$$

For convenience, we shall set $\mu_{t}=\mu_{0}$ for t⩽0 and $\mu_{t}=\mu_{T}$ for t⩾T as well as $v_{t}=0$ for t∉[0,T] . We define the function $\eta :(x,t)\in M\times \mathbb{R}\mapsto \frac{1}{2}\int_{M}W(x,y)\;d\mu_{t}(y)$ for which

$$\partial_{t}\eta (t,x)=\left\{ \begin{array}{ll} 0 & \;\text{if }t\notin [0,T],\\ \frac{1}{2}\int_{M}\langle D_{y}W(x,y),v_{t}(y)\rangle \;d\mu_{t}(y) & \;\text{else}, \end{array}\right.$$

in the distributional sense. Using the mollifier $g_{\epsilon }$ , as described in [71, ch. C.5], one can smooth out η in the time direction by setting

$$\begin{array}{r} \eta_{\epsilon }(t,x)≔\int_{\mathbb{R}}\eta (\tau ,x)g_{\epsilon }(t-\tau )\;d\tau . \end{array}$$

By [71, ch. C.5, Theorem 7 (iii)], we have that $\eta_{\epsilon }\to \eta$ pointwise, and with the use of the dominated convergence theorem with the upper bound $|\eta_{\epsilon }|⩽\sup_{(x,y)\in M\times M}|W(x,y)|<\infty$ , we calculate

$$\begin{array}{r} E(\mu_{T})-E(\mu_{0})=\int_{M}\eta \;d\mu_{T}-\int_{M}\eta \;d\mu_{0}=\lim_{\epsilon \to 0}\;\int_{M}\eta_{\epsilon }\;d\mu_{T}-\int_{M}\eta_{\epsilon }\;d\mu_{0}. \end{array}$$

We further have that

$$\begin{array}{rl} +\infty & >\frac{1}{2}\int_{0}^{T}\int_{M}\int_{M}\langle D_{y}W(y,x),v_{t}(y)\rangle \;d\mu_{t}(y)\;d\mu_{t}(x)\;dt\\ & \overset{\left(**\right)}{=}\lim_{\epsilon \to 0}\;\frac{1}{2}\int_{0}^{T}\int_{\mathbb{R}}\int_{M}\int_{M}\langle D_{y}W(x,y),v_{t}\rangle \;d\mu_{t}(y)g_{\epsilon }(t-\tau )(t)\;d\tau \;d\mu_{t}(x)\;dt\\ & \overset{\left(*\right)}{=}-\lim_{\epsilon \to 0}\;\int_{0}^{T}\int_{M}\int_{\mathbb{R}}\eta (\tau ,x)\partial_{\tau }g_{\epsilon }(t-\tau )\;d\tau \;d\mu_{t}(x)\;dt\\ & =\lim_{\epsilon \to 0}\;\int_{0}^{T}\int_{M}\int_{\mathbb{R}}\eta (\tau ,x)\partial_{t}g_{\epsilon }(t-\tau )\;d\tau \;d\mu_{t}(x)\;dt=\lim_{\epsilon \to 0}\;\int_{0}^{T}\int_{M}\partial_{t}\eta_{\epsilon }(t,x)\;d\mu_{t}(x)\;dt, \end{array}$$

where for (∗) we use the definition of the distributional derivative and rearrange the integral using the Fubini–Tonelli theorem. To prove (∗∗) , we need to define a piecewise constant approximation of $\mu_{t}$ . We fix a N∈ℕ $\tau =\frac{T}{N}$ and set for k∈{1,N}

$$\begin{array}{r} {\bar{\mu }}_{t}≔\mu_{k\tau }\;\text{for }t\in [k\tau ,(k+1)\tau ),\;{\bar{\mu }}_{T}≔\mu_{T}. \end{array}$$

Since $\mu_{t}$ is 1 -Lipschitz, we have $W_{2}(\mu_{t},{\bar{\mu }}_{t})⩽\tau$ for all t∈[0,T] . Then, we estimate

|∫0T∫M∫M⟨DyW(x,y),vt(y)⟩dμt(y)dμt(x)dt−∫0T∫M∫M⟨DyW(x,y),vt(y)⟩dμt(y)dμ¯t(x)dt|⩽∫0T∫M×M∫M|⟨DyW(x1,y),vt(y)⟩−⟨DyW(x2,y),vt(y)⟩|dμt(y)dπt(x1,x2)dt⩽∫0T∫M×M∫M|DyW(x1,y)−DyW(x2,y)|∗|vt(y)|dμt(y)dπt(x1,x2)dt⩽C∫0T∫M×M∫M|x1−x2||vt(y)|dμt(y)dπt(x1,x2)dt=C∫0T(∫M×M|x1−x2|dπt(x1,x2))(∫M|vt(y)|dμt(y))dt⩽C(∫0TW22(μt,μ¯t)dt∫0T∫M|vt(y)|2dμt(y)dt)1/2(A 11)=C(T∫0TW22(μt,μ¯t)dt)1/2⩽C(T∫0Tτ2dt)1/2=CT2N,

where $\pi_{t}\in P(M\times M)$ is the optimal transport plan between $\mu_{t}$ and ${\bar{\mu }}_{t}$ and $|\cdot {|}_{*}$ denotes the dual norm of |⋅| . (For more details on the static formulation of Wasserstein distances via optimal transport plans, we refer to [53, ch. 6]). We can argue similarly in the mollified case:

|∫0T∫M∫R∫M⟨DyW(x,y),vτ(y)⟩dμτ(y)gϵ(t−τ)dτdμt(x)dt−∫0T∫M∫R∫M⟨DyW(x,y),vτ(y)⟩dμτ(y)gϵ(t−τ)dτdμ¯t(x)dt|⩽∫0T∫M×M∫R∫M|⟨DyW(x1,y),vτ(y)⟩−⟨DyW(x2,y),vτ(y)⟩|dμτ(y)gϵ(t−τ)dτdπt(x1,x2)dt⩽∫0T∫M×M∫R∫M|DyW(x1,y)−DyW(x2,y)|∗|vτ(y)|dμτ(y)gϵ(t−τ)dτdπt(x1,x2)dt⩽C∫0T∫M×M∫R∫M|x1−x2||vτ(y)|dμτ(y)gϵ(t−τ)dτdπt(x1,x2)dt=C∫0T(∫M×M|x1−x2|dπt(x1,x2))(∫R∫M|vτ(y)|dμτ(y)gϵ(t−τ)dτ)dt⩽C∫0TW2(μt,μ¯t)∫R∫M|vτ(y)|dμτ(y)gϵ(t−τ)dτdt⩽CTN∫0T∫R∫M|vτ(y)|dμτ(y)gϵ(t−τ)dτdt=CTN∫R∫M|vτ(y)|dμτ(y)∫0Tgϵ(t−τ)dtdτ≤CTN∫R∫M|vτ(y)|dμτ(y)dτ=CTN∫0T∫M|vτ(y)|dμτ(y)dτ(A 12)≤CTN∫0T(∫M|vτ(y)|2dμτ(y))1/2dτ⩽CT2N.

We denote $\tilde{C}=\sup_{(x,y)\in M\times M}|D_{y}W(x,y){|}_{*}<+\infty$ and combine equations (A 11) and (A 12) to estimate

$$\begin{array}{rl} & |\int_{0}^{T}\int_{M}\int_{M}\left\langle D_{y}W(x,y),v_{t}(y)\right\rangle d\mu_{t}(y)d\mu_{t}(x)dt\\ & -\int_{0}^{T}\int_{M}\int_{R}\int_{M}\left\langle D_{y}W(x,y),v_{\tau }(y)\right\rangle d\mu_{\tau }(y)g_{\epsilon }(t-\tau )d\tau \;d\mu_{t}(x)dt|\\ & ⩽2C\frac{T^{2}}{N}+|\int_{0}^{T}\int_{M}\underset{:=f(x,t)}{\underbrace{\int_{M}\left\langle D_{y}W(x,y),v_{t}(y)\right\rangle d\mu_{t}(y)}}d{\bar{\mu }}_{t}(x)dt-\\ & \int_{0}^{T}\int_{M}\underset{:=f_{\epsilon }(x,t)}{\underbrace{\int_{R}\int_{M}\left\langle D_{y}W(x,y),v_{\tau }(y)\right\rangle d\mu_{\tau }(y)g_{\epsilon }(t-\tau )d\tau }}d{\bar{\mu }}_{t}(x)dt|\\ & ⩽2C\frac{T^{2}}{N}+\sum_{i=1}^{N}\int_{(i-1)\tau +\epsilon }^{i\tau -\epsilon }\int_{M}\left|f-f_{\epsilon }\right|d{\bar{\mu }}_{t}\;dt+\int_{(i-1)\tau }^{(i-1)\tau +\epsilon }\int_{M}\left|f-f_{\epsilon }\right|d{\bar{\mu }}_{t}\;dt+\\ & \int_{i\tau -\epsilon }^{i\tau }\int_{M}\left|f-f_{\epsilon }\right|d{\bar{\mu }}_{t}\;dt\\ & ⩽2C\frac{T^{2}}{N}+\sum_{i=1}^{N}\int_{(i-1)\tau +\epsilon }^{i\tau -\epsilon }\int_{M}\left|f-f_{\epsilon }\right|d{\bar{\mu }}_{t}\;dt+\int_{(i-1)\tau }^{(i-1)\tau +\epsilon }\int_{M}2\tilde{C}\;d{\bar{\mu }}_{t}\;dt+\int_{i\tau -\epsilon }^{i\tau }\int_{M}2\tilde{C}\;d{\bar{\mu }}_{t}\;dt\\ & ⩽2C\frac{T^{2}}{N}+\sum_{i=1}^{N}\int_{(i-1)\tau +\epsilon }^{i\tau -\epsilon }\int_{M}\left|f-f_{\epsilon }\right|d{\bar{\mu }}_{t}\;dt+4N\epsilon \tilde{C}⩽\frac{\delta }{3}+\sum_{i=1}^{N}\frac{\delta }{3N}+\frac{\delta }{3}, \end{array}$$

where, first, N is chosen such that $N⩾6C\frac{T^{2}}{\delta }$ and, second, ϵ such that $\epsilon ⩽\frac{\delta }{12N\tilde{C}}$ and for each i∈{1,...,N} , it holds $\int_{(i-1)\tau +\epsilon }^{i\tau -\epsilon }\int_{M}|f-f_{\epsilon }|\;d{\bar{\mu }}_{t}\;dt⩽\frac{\delta }{3N}$ (by lemma A.3). Therefore (∗∗) is proven.

Finally, by lemma A.4, we obtain $n_{\epsilon }\in C^{1}(M\times [0,T])$ that we can use as a test function in equation (A 3) and send ϵ→0 to obtain

$\begin{array}{rl} E\left(\mu_{T}\right)-E\left(\mu_{0}\right) & =\int_{M}\eta \;d\mu_{T}-\int_{M}\eta \;d\mu_{0}=\int_{0}^{T}\int_{M}\partial_{t}\eta \;d\mu_{t}+\int_{M}\left\langle D\eta ,v_{t}\right\rangle d\mu_{t}\;dt\\ & =\int_{0}^{T}\int_{M\times M}\left\langle D_{x}W(x,y),v_{t}(x)\right\rangle d\mu_{t}(x)d\mu_{t}(y)dt. \end{array}$

∎

**Lemma A.3**. *Let* f:M×[0,T]→ℝ *be Borel measurable and* μ∈P(M) *with*

$$\int_{a}^{b}\int_{M}|f|\;d\mu \;dt<\infty \;\text{for }0⩽a<b⩽T.$$

*For*

$$\mu_{a}^{b}(A)≔\mu ⊗L(a,b)$$

*it holds*

$$\|f_{\epsilon }{\|}_{L^{1}(\mu_{a+\epsilon }^{b-\epsilon })}⩽\|f{\|}_{L^{1}(\mu_{a}^{b})}\;\text{and}\;f_{\epsilon }\to f\;\text{in }L^{1}(\mu_{a+\epsilon }^{b-\epsilon }).$$

*Proof*. We adapt [71, ch. 5, Theorem 7] to our case and start by showing

$$\begin{array}{rl} \|f_{\epsilon }{\|}_{L^{1}(\mu_{a+\epsilon }^{b-\epsilon })}⩽ & \int_{M}\int_{a+\epsilon }^{b-\epsilon }\int_{a}^{b}|f(x,\tau )|g_{\epsilon }(t-\tau )\;d\tau \;dt\;d\mu (x)\\ = & \int_{M}\int_{a}^{b}|f(x,\tau )|\int_{a+\epsilon }^{b-\epsilon }g_{\epsilon }(t-\tau )\;dt\;d\tau \;d\mu (x)=\int_{M}\int_{a}^{b}|f(x,\tau )|\;d\tau \;d\mu (x)=\|f{\|}_{L^{1}(\mu_{a}^{b})}. \end{array}$$

We approximate f in $L^{1}(\mu_{a}^{b})$ by $\gamma \in C_{c}(M\times [a,b])$ (see [70, Proposition 7.9]) and calculate

$$\begin{array}{rl} \|f-f_{\epsilon }{\|}_{L^{1}(\mu_{a+\epsilon }^{b-\epsilon })}⩽ & \|f-\gamma {\|}_{L^{1}(\mu_{a+\epsilon }^{b-\epsilon })}+\|\gamma -\gamma_{\epsilon }{\|}_{L^{1}(\mu_{a+\epsilon }^{b-\epsilon })}+\|\gamma_{\epsilon }-f_{\epsilon }{\|}_{L^{1}(\mu_{a+\epsilon }^{b-\epsilon })}\\ ⩽ & 2\|f-\gamma {\|}_{L^{1}(\mu_{a}^{b})}+\|\gamma -\gamma_{\epsilon }{\|}_{L^{1}(\mu_{a+\epsilon }^{b-\epsilon })}. \end{array}$$

From [71, ch. C.5, Theorem 7], we know that $\gamma_{\epsilon }\to \gamma$ for all (x,t)∈M×[a,b] because γ is continuous. Choosing γ such that $\|f-\gamma {\|}_{L^{1}(\mu_{a}^{b})}<\delta$ and using the dominated convergence theorem, we get ${\mathrm{lim sup}}_{\epsilon \to 0}\|f-f_{\epsilon }{\|}_{L^{1}(\mu_{a+\epsilon }^{b-\epsilon })}⩽2\delta$ . As δ can be chosen arbitrarily small, we obtain convergence.∎

**Lemma A.4**. *We have* $\eta_{\epsilon }\in C^{1}(M\times [0,T])$ .

*Proof*. Let $\gamma :V\subset {\mathbb{R}}^{d}\to U(x)$ be a smooth local chart for an open set U(x) containing x . Then, since $W(x,y)\in C^{1}(M\times M)$ , the function $z\mapsto \int_{M}\partial_{z_{i}}W(\gamma (z),y)\;d\mu_{\tau }$ is continuous in z and the product

$$\left(z,t)\mapsto \int_{M}\partial_{z_{i}}W(\gamma (z),y)\;d\mu_{\tau }(y)g_{\epsilon }(t-\tau \right)$$

is continuous on V×ℝ . Taking any sequence $(z_{n},t_{n})\to \left(z,t\right)$ , we can use the dominated convergence theorem to obtain

$$\begin{array}{rl} \lim_{n\to \infty }\partial_{t}\eta_{\epsilon }(\gamma (z_{n}),t_{n}) & =\lim_{n\to \infty }\int_{R}\int_{M}\partial_{z_{i}}W(\gamma (z_{n}),y)\;d\mu_{\tau }(y)g_{\epsilon }(t_{n}-\tau )\;d\tau \\ & =\int_{R}\int_{M}\partial_{z_{i}}W(\gamma (z),y)\;d\mu_{\tau }(y)g_{\epsilon }(t-\tau )\;d\tau =\partial_{t}\eta_{\epsilon }(\gamma (z),t). \end{array}$$

An upper bound is given by the function $\sup_{V\times W}\;\partial_{z_{i}}W(\gamma (z),y)\chi_{\left[\inf_{n}\;t_{n}-\epsilon ,\sup_{n}\;t_{n}+\epsilon \right]}(\tau )$ . Thus, $\partial_{t}\eta_{\epsilon }(\gamma (z),t)$ is continuous in V×[0,T] . With the same argument, a similar statement can be shown for

$$\begin{array}{r} \partial_{t}\eta_{\epsilon }(x,t)=\int_{R}\int_{M}W(x,y)\;d\mu_{\tau }(y)\partial_{t}g_{\epsilon }(t-\tau )(t)\;d\tau . \end{array}$$

By [72, Theorem 2.8], it follows that $\eta_{\epsilon }(t,\gamma (z))\in C^{1}(V\times [0,T])$ and since the local chart was chosen arbitrarily $\eta_{\epsilon }\in C^{1}(M\times [0,T])$ .∎

## Appendix B. Spherical coordinates

For many computations in §4, we use spherical coordinates. Up to small notational changes, we use the definition provided in [73]. We define the coordinate transform $X_{n}:\varphi \in [0,\pi ]{,}^{n-2}\times [0,2\pi ]\to S^{n-1}$ for $\varphi \in [0,\pi ]{,}^{n-2}\times \left[0,2\pi \right]$ as

$$X_{n}(\varphi )=\cos\left(\varphi_{1}\right)e_{1}+\sum_{i=2}^{n-1}\cos\left(\varphi_{i}\right)\prod_{j=1}^{i-1}\sin\left(\varphi_{j}\right)e_{i}+\prod_{j=i}^{n-1}\sin\left(\varphi_{i}\right)e_{n}.$$

Here and in the following, $e_{i}\in {\mathbb{R}}^{n}$ denotes the i th standard basis vector.

The Jacobian determinant is given by

$$\begin{array}{r} JX_{n}(\varphi )=\prod_{i=1}^{n-2}\sin^{n-1-i}(\varphi_{i}). \end{array}$$

To highlight the recursive character of $X_{n}$ with respect to n , we further note that

$$\begin{array}{r} X_{n}(\varphi {)}_{\hat{1}}=\sin(\varphi_{1})\;X{\;}_{n-1}(\varphi_{\hat{1}})\;\text{and}\;JX_{n}(\varphi )=\sin^{n-2}(\varphi_{1})JX_{n-1}(\varphi_{\hat{1}}), \end{array}$$

where the index $\hat{1}$ denotes that we drop the first element, i.e. for $\varphi \in {\mathbb{R}}^{n-1}$ , $\varphi_{\hat{1}}=\sum_{i=2}^{n-1}\varphi_{i}\;e_{i-1}$ . A practical consequence of this property is the recursive computation formula for the Hausdorff measure of the n -dimensional sphere.

**Lemma B.1**. Denote $|S^{n-1}|:=H^{n}(S^{n-1})$ . For n⩾2 , it holds that

$$\begin{array}{r} |S^{n-1}|=|S^{n-2}|\int_{0}^{\pi }\sin^{n-2}\;\varphi \;\;d\varphi . \end{array}$$

*Proof*. For n=2 , the proof follows from a simple computation and the fact that $|S^{0}|=2$ and $|S^{1}|=2\pi$ . For n>2 , we have

$$\begin{array}{rl} |S^{n-\;1}| & =\int_{\left[0,\pi {]}^{n-2}\times [0,2\pi \right]}JX_{n}(\varphi )\;d\varphi =\int_{0}^{\pi }\sin^{n-2}\varphi_{1}\;JX_{n-1}(\varphi_{\hat{1}})\;d\varphi \\ & =\int_{0}^{\pi }\sin^{n-2}\varphi \;d\varphi \;\int_{\left[0,\pi {]}^{n-3}\times [0,2\pi \right]}JX_{n-1}(\psi )\;d\psi =|{§}^{n-2}|\int_{0}^{\pi }\sin^{n-2}\varphi \;d\varphi , \end{array}$$

where we use the recursive property of the Jacobian determinant. ∎

### B.1. Definition using Givens rotations

Spherical coordinates can equivalently be defined using Givens rotations (see e.g. [74, ch. 5.1.8]). A Givens rotation for an angle φ∈[0,2π) and indices i,j⩽n with i≠j is determined by the rotation matrix $G(i,j,\varphi )\in {\mathbb{R}}^{n\times n}$ :

$$\begin{array}{r} G(i,j,\varphi {)}_{k,l}=\left\{ \begin{array}{ll} \cos(\varphi ) & \;\text{if }k=l=i\text{ or }k=l=j,\\ 1 & \;\text{if }k=l\neq i\text{ and }k=l\neq j,\\ \sin(\varphi ) & \;\text{if }k=i,l=j,\\ -\sin(\varphi ) & \;\text{if }k=j,l=i,\\ 0 & \;\text{otherwise.} \end{array}\right. \end{array}$$

Applying $G(i,j,\varphi {)}^{T}$ to a vector $x\in R^{n}$ corresponds to a counterclockwise rotation of x by the angle φ in the (i,j) -plane. For a given vector of angles $\varphi \in [0,\pi ]{,}^{n-2}\times \left[0,2\pi \right]$ , we can thus construct the matrix

(B 1)
$$\begin{array}{lrr} & \begin{array}{rl} R(\varphi )=G(n-1,n,\varphi_{n-1})\circ \ldots & \circ G(2,3,\varphi_{2})\\ & \circ G(1,2,\varphi_{1})\circ G(2,3,\varphi_{2}{)}^{T}\circ \cdots \circ G(n-1,n,\varphi_{n-1}{)}^{T}. \end{array} & \end{array}$$

The rotation matrix R(φ) can be written as a two-dimensional rotation of angle $\varphi_{1}$ in the $\left(e_{1},X_{n-1}(\varphi_{\hat{1}})\right)$ -plane, as the following lemma shows.

**Lemma B.2**. *Let* R(φ) *be the rotation matrix as described in* *equation (B 1)*. *Then, it holds that*

$$\begin{array}{r} R(\varphi )=U\;G(1,2,\varphi_{1})\;U{,}^{T}, \end{array}$$

*with* $UU^{T}=\text{Id}$ *,* $U_{1,\cdot }=e_{1}$ *and* $U_{2,\cdot }=(0,\;X{,}_{n-1}(\varphi_{\hat{1}}){)}^{T}$ *.*

*Proof*. For n=2 , the statement can be verified by inserting U=Id⁡ and the definition of R(φ) . For n>2 , we define

$$\begin{array}{r} U=G(2,3,\varphi_{2})\circ \cdots \circ G(n-1,n,\varphi_{n-1}). \end{array}$$

With this choice of U , R(φ) has the claimed form and $UU^{T}=\mathrm{Id}$ due to the orthogonality of Givens matrices. It remains to show that the first two rows of U fulfil $U_{1,\cdot }=e_{1}$ and $U_{2,\cdot }=(0,\;X{,}_{n-1}(\varphi_{\hat{1}}){)}^{T}$ . For n=3 , U reduces to

$$\begin{array}{r} U=G(2,3,\varphi_{2})=\left(\begin{array}{ccc} 1 & 0 & 0\\ 0 & \cos\;\varphi_{2} & \sin\;\varphi_{2}\\ 0 & -\sin\;\varphi_{2} & \cos\;\varphi_{2} \end{array}\right), \end{array}$$

and clearly, $U_{1,\cdot }=e_{1}$ and $U_{2,\cdot }=(0,\cos\;\varphi_{2},\sin\;\varphi_{2}{)}^{T}=(0,X_{2}(\varphi_{2}){)}^{T}$ . For n>3 , the proof follows from induction over n .∎

**Corollary B.3**. *Let* $x=X_{n}(\varphi )$ *,* $\tilde{x}=\left(0,X_{n-1}(\varphi_{\hat{1}})\right)$ *then*

$$\begin{array}{rl} R(\varphi {)}^{T}y=y-\left(y\cdot e_{1}\right)e_{1}-(y\cdot \tilde{x})\tilde{x} & +\left(\cos\left(\varphi_{1}\right)\left(y\cdot e_{1}\right)-\sin\left(\varphi_{1}\right)(y\cdot \tilde{x})\right)e_{1}\\ + & \left(\sin\left(\varphi_{1}\right)\left(y\cdot e_{1}\right)+\cos\left(\varphi_{1}\right)(y\cdot \tilde{x})\right)\tilde{x} \end{array}$$

*In particular, if* $y\cdot e_{1}=0$ *it holds that*

$$\begin{array}{rl} R(\varphi {)}^{T}y=y & -(y\cdot \tilde{x})\;\tilde{x}+(y\cdot \tilde{x})\left(-\sin(\varphi_{1})\;e{,}_{1}+\cos(\varphi_{1})\;\tilde{x}\right). \end{array}$$

With the above results, we obtain

$$\begin{array}{r} X_{n}(\varphi )=R(\varphi {)}^{T}e_{1}, \end{array}$$

and since Givens matrices are orthonormal, it also holds that

$$\begin{array}{r} R(\varphi )X_{n}(\varphi )=e_{1}. \end{array}$$

We can also therefore consider rotated spherical coordinates

$$\begin{array}{r} X_{n}^{\theta }(\varphi )=R(\theta {)}^{T}X_{n}(\varphi ) \end{array}$$

for a reference point $x=X_{n}(\theta )$ , with the same Jacobian determinant as before, i.e. $JX_{n}^{\theta }(\varphi )=JX_{n}(\varphi )$ .

## Appendix C. Proofs for Section 4

### C.1. Proof of Lemma 4.10

**Lemma 4.7 (cont.)** Let n>2 . The uniform distribution $\mu =\frac{1}{\left|S^{n-1}\right|}H^{n}$ is a stationary point of E if and only if all eigenvalues $\{\lambda {,}_{i}{\}}_{i=1}^{n}$ of D have the same absolute value, i.e. $|\lambda {,}_{i}|=\lambda$ for some λ∈ℝ .

*Proof*. The proof for n>2 uses the same arguments as for n=2 ; however, the rotation corresponding to a translation of the angle in two dimensions is technically more complicated. We use the notation and techniques from appendix B (spherical coordinates $X_{n}$ and rotations R ).

Again, we first fix $x\in S^{n-1}$ and consider the integral

$$\begin{array}{r} \int_{S^{n-1}}e^{x\cdot Dy}P_{x}^{⟂}Dy\;\;dH{,}^{n}(y)=(*). \end{array}$$

Similarly to the two-dimensional case, we choose $\varphi \in [0,\pi ]{,}^{n-2}\times \left[0,2\pi \right]$ such that

$$\begin{array}{r} X_{n}(\varphi )=\frac{Dx}{\left\|Dx\right\|}, \end{array}$$

and therefore also

$$\begin{array}{r} R(\varphi )Dx=\|Dx\|R(\varphi )R(\varphi {)}^{T}e_{1}=\|Dx\|e_{1}, \end{array}$$

where $e_{1}=(1,0,\ldots ,0{)}^{T}\in {\mathbb{R}}^{n}$ denotes the first standard basis vector. We rewrite the integral using rotated spherical coordinates and substitute it into the above identity to obtain

$$\begin{array}{rl} (*)= & \int_{\left[0,\pi {]}^{n-2}\times [0,2\pi \right]}e^{x\cdot DR(\varphi {)}^{T}X_{n}(\theta )}P_{x}^{⊥}DR(\varphi {)}^{T}X_{n}(\theta )\;JX_{n}(\theta )\;\;d\theta \\ = & \int_{\left[0,\pi {]}^{n-2}\times [0,2\pi \right]}e^{\left\|Dx\|\;\cos(\theta_{1}\right)}(DR(\varphi {)}^{T}X_{n}(\theta )-\|Dx\|\;\cos(\theta_{1})\;x)\;JX_{n}(\theta )\;\;d\theta , \end{array}$$

where $JX_{n}$ denotes the Jacobian determinant of $X_{n}$ . To reduce the above integral over the vector θ to an integral over only the first component $\theta_{1}$ , we write

$$\begin{array}{rl} R(\varphi {)}^{T}X_{n}(\theta ) & =\cos(\theta_{1})\;R(\varphi {)}^{T}e_{1}+R(\varphi {)}^{T}\left(\begin{array}{c} 0\\ X_{n}(\theta {)}_{\hat{1}} \end{array}\right)\\ & =\cos(\theta_{1})\frac{Dx}{\left\|Dx\right\|}+\sin(\theta_{1})\;R(\varphi {)}^{T}\left(\begin{array}{c} 0\\ X_{n-1}(\theta_{\hat{1}}) \end{array}\right), \end{array}$$

where the subscript $\hat{1}$ denotes that we neglect the first component. Inserting this into (∗) , we get

$$\begin{array}{rl} \left(*\right) & =\left(D^{2}x/\|Dx\|-\|Dx\|x\right)\int_{0}^{\pi }e^{\left\|Dx\|\;\cos(\theta_{1}\right)}\cos(\theta_{1})\sin^{n-2}(\theta_{1})\;\;d\theta {\;}_{1}\\ & \;+\int_{0}^{\pi }e^{\left\|Dx\|\;\cos(\theta_{1}\right)}\sin^{n-1}(\theta_{1})\;DR(\theta {)}^{T}\underset{=0}{\underbrace{\int_{S^{n-2}}\left(\begin{array}{c} 0\\ z \end{array}\right)\;dH^{n-1}(z)}}\;\;d\theta {\;}_{1}\\ & =C(n,\|Dx\|)\left(D^{2}x/\|Dx\|-\|Dx\|x\right), \end{array}$$

and due to the symmetry of sine and cosine, we have that C(n,‖Dx‖)>0 for any n⩾2 , ‖Dx‖>0 . We can thus deduce that (∗)=0 if and only if x is an eigenvector of $D^{2}$ , exactly as in the case n=2 . This holds true for μ -almost all $x\in S^{n-1}$ if and only if all eigenvalues of D have the same absolute value, which then automatically yields $\;dE_{D}(\mu ,V)=0$ .

Again, it remains to show that this is also necessary. Without loss of generality, we assume $|\lambda {,}_{1}|>\left|\lambda {,}_{2}\right|$ and $\lambda {,}_{1}$ and $\lambda {,}_{2}$ to be the eigenvalues of largest, respectively second largest, absolute value corresponding to the eigenvectors $z_{1}$ , respectively $z_{2}$ .

From here, the strategy is the exact same as in the two-dimensional case, which we restate here for completeness. The factor $\left(D^{2}x/\|Dx\|-\|Dx\|x\right)\cdot z_{2}$ is strictly negative on the set

$$\begin{array}{r} A=\{x\in S^{n-1}\;|\;(x\cdot z_{1})\in (|\lambda {,}_{2}/\lambda {,}_{1}|,1),\;(x\cdot z_{2})>0\}. \end{array}$$

Since μ(A)>0 , we can find a Lipschitz continuous V such that $V\cdot z_{1}=0$ for μ -a.e. on $S^{n-1}$ and

$$\begin{array}{r} V(x)\cdot z_{2}=\left\{ \begin{array}{ll} >0\; & \text{for a.e. }x\in A,\\ =0\; & \text{for a.e. }x\in S^{n-1}\setminus A. \end{array}\right. \end{array}$$

For all such V , it holds that $\;dE_{D}(\mu ,V)>0$ , which concludes the proof.∎

### C.2. Proof of Lemma 4.12

**Lemma 4.8 (cont.)** Let n⩾2 , and $\mu_{0}=\frac{1}{\left|S^{n-1}\right|}H^{n}$ . Then, it holds that

(C 1)∫Sn−1ex⋅yx dμ0(x)=C1y

*for any* $y\in S^{n-1}$ *, where the constant* $C_{1}$ *is positive and depends only on the dimension* n .

*Proof*. The proof for n>2 goes along the lines of the proof for n=2 . However, the rotation corresponding to a translation of the angle in two dimensions is technically more complicated in higher dimensions. For an introduction to rotated spherical coordinates used in this proof, we refer the reader to appendix B.

We first fix $y\in S^{n-1}$ and choose $\theta \in {\mathbb{R}}^{n-1}$ such that $y=X_{n}(\theta )$ . We proceed to write the integral using rotated spherical coordinates $x=X_{n}^{\theta }(\varphi )$ and obtain

$$\int_{S^{n-1}}e^{x\cdot y}x_{j}\;d\mu_{0}(x)=\frac{1}{\left|S^{n-1}\right|}\int_{\left[0,\pi {]}^{n-2}\times [0,2\pi \right]}e^{X_{n}^{\theta }(\varphi )\cdot X_{n}(\theta )}{\left(X_{n}^{\theta }(\varphi )\right)}_{i}JX_{n}(\varphi )\;d\varphi =(*).$$

Substituting the expressions for $X_{n}$ and $X_{n}^{\theta }$ yields

$$\begin{array}{rl} X_{n}^{\theta }(\varphi )\cdot X_{n}(\theta ) & =R(\theta {)}^{T}X_{n}(\varphi )\cdot X_{n}(\theta )=X_{n}(\varphi )\cdot R(\theta )X_{n}(\theta )=X_{n}(\varphi )\cdot e_{1}=\cos(\varphi_{1}). \end{array}$$

In addition, we note that we can write any $x=x_{1}e_{1}+(0,x_{2},\ldots ,x_{n}{)}^{T}$ and see that

$$\begin{array}{rl} X_{n}^{\theta }(\varphi )=R(\theta {)}^{T}X_{n}(\varphi )= & \;R(\theta {)}^{T}\cos(\varphi_{1})e_{1}+R(\theta {)}^{T}\left(\begin{array}{c} 0\\ X_{n}(\varphi {)}_{\hat{1}} \end{array}\right)\\ = & \;\cos(\varphi_{1})\;y+\sin(\varphi_{1})R(\theta {)}^{T}\left(\begin{array}{c} 0\\ X_{n-1}(\varphi_{\hat{1}}) \end{array}\right), \end{array}$$

where $e_{1}=(1,0,\ldots ,0{)}^{T}\in {\mathbb{R}}^{n}$ denotes the first standard basis vector. Substituting the above equality into the integral, we derive

$$\begin{array}{rl} (*)= & \frac{1}{|S^{n-1}|}\int_{\left[0,\pi {]}^{n-2}\times [0,2\pi \right]}e^{\cos(\varphi_{1})}{\left[\cos(\varphi_{1})y+\sin(\varphi_{1})R(\theta {)}^{T}\left(\begin{array}{c} 0\\ X_{n-1}(\varphi_{\hat{1}}) \end{array}\right)\right]}_{j}\;JX_{n}(\varphi )\;d\varphi \\ = & y_{i}\;\frac{|S^{n-2}|}{|S^{n-1}|}\int_{0}^{\pi }e^{\cos\varphi }\cos\varphi \sin^{n-2}\varphi \;d\varphi \\ & +\frac{1}{|S^{n-1}|}\int_{0}^{\pi }e^{\cos\varphi }\sin^{n-1}\varphi {\left[R(\theta {)}^{T}\underset{=0}{\underbrace{\int_{S^{n-2}}\left(\begin{array}{c} 0\\ z \end{array}\right)\;dH^{n-1}(z)}}\right]}_{j}\;d\varphi . \end{array}$$

The proof now follows from choosing the constant:

$$\begin{array}{r} C_{1}=\frac{\left|S^{n-2}\right|}{\left|S^{n-1}\right|}\int_{0}^{\pi }e^{\cos\;\varphi }\cos\;\varphi \;\sin^{n-2}\;\varphi \;\;d\varphi =\frac{\left|S^{n-2}\right|}{\left|S^{n-1}\right|}\int_{0}^{\pi /2}\sin^{n-2}\;\varphi \;\cos\;\varphi \;\sinh(\cos\;\varphi )\;\;d\varphi , \end{array}$$

which is positive for all n⩾2 since the function t↦tsinh⁡t is positive for t>0 and both sine and cosine are positive for φ∈(0,π/2) .∎

### C.3. Proof of Lemma 4.13

**Lemma 4.9 (cont.)** Let n⩾2 , and $\mu_{0}=\frac{1}{\left|S^{n-1}\right|}H^{n}$ . Then, for all $y\in S^{n-1}$ and 1⩽i⩽n , it holds that

(C 2)∫Sn−1ex⋅yxi2 dμ0(x)=C2yi2+C3,

*where the constants* $C_{2}$ *and* $C_{3}$ *are positive and depend only on the dimension* n .

*Proof*. Using the same arguments as in the previous proof, we obtain

∫0πex⋅yxj2 dμ0(x)=yj2|Sn−2||Sn−1|∫Sn−1ecos⁡φcos2⁡φsinn−2⁡φ dφ(C 3)+1|Sn−1|∫0πecos⁡φsinn⁡φ∫Sn−2[R(θ)T(0z)]j2 dHn−1(z)dφ,

where the mixed term containing $x_{i}y_{i}$ vanishes due to symmetry. Since the second term still depends on y due to the rotation, we write $\tilde{y}=\left(0,X_{n-1}(\theta_{\hat{1}})\right)$ and decompose $\tilde{z}=\left(0,z\right)$ into its rotation-invariant and rotation-variant part. More precisely, we use corollary B.3 to get

$$\begin{array}{r} R(\theta {)}^{T}\tilde{z}=\tilde{z}-(\tilde{y}\cdot \tilde{z})\tilde{y}+(\tilde{y}\cdot \tilde{z})\left[-\sin(\theta_{1})\;e{,}_{1}+\cos(\theta_{1})\;\tilde{y}\right] \end{array}$$

and thus

$$\begin{array}{r} {\left[R(\theta {)}^{T}\tilde{z}\right]}^{2}=(\tilde{z}-(\tilde{y}\cdot \tilde{z})\tilde{y}{)}^{2}+(\tilde{y}\cdot \tilde{z}{)}^{2}(\sin^{2}(\theta_{1})\;e{,}_{1}+\cos^{2}(\theta_{1})\;\tilde{y}{,}^{2})+2\;\cos(\theta_{1})(\tilde{z}-(\tilde{y}\cdot \tilde{z})\;\tilde{y})(\tilde{y}\cdot \tilde{z})\;\tilde{y}. \end{array}$$

Making use of the trigonometric identity $\cos^{2}(\theta_{1})+\sin^{2}(\theta_{1})=1$ , we get

[R(θ)Tz~]2=z~2+(y~⋅z~)2e1+2(y~⋅z~)2y~2−2(y~⋅z~)z~y~+2cos⁡(θ1)(z~−(y~⋅z~)y~)(y~⋅z~)y~−(y~⋅z~)2(cos2⁡(θ1)e1+sin2⁡(θ1)y~2),(C 4)=z~2+(y~⋅z~)2(e1−y2)+2(cos⁡(θ1)−1)(z~−(y~⋅z~)y~)(y~⋅z~)y~,

where in the last step, we use the fact that $y^{2}=\cos^{2}\;\theta_{1}e_{1}+\sin^{2}\;\theta_{1}{\tilde{y}}^{2}$ . To prove that the integral over the expression in equation (C 4) can be written as claimed, we observe that for all j=2,…,n

$$\begin{array}{r} \int_{S^{n-2}}{\tilde{z}}_{j}^{2}\;\;dH{,}^{n-1}(z)=:\tilde{C}, \end{array}$$

where $\tilde{C}$ is positive and depends only on n , and therefore, also $\int_{S^{n-2}}(\tilde{z}\cdot \tilde{y}{)}^{2}\;\;dH{,}^{n-1}(z)=\tilde{C}\;\|\tilde{y}{\|}^{2}=\tilde{C}$ . With this, we derive that

(C 5)[∫Sn−2z~2+(y~⋅z~)2(e1−y2)dHn−1(z)]j=C~(1−yj2)

for all j=1,…,n and it remains to show that for any 1⩽j⩽n

(C 6)∫Sn−2[(z~−(y~⋅z~)y~)(y~⋅z~)y~]jdH n−1(z)=0.

The case j=1 is trivial as ${\tilde{y}}_{1}={\tilde{z}}_{1}=0$ . For 2⩽j⩽n , we write out the integrand and obtain

$$\begin{array}{rl} & [(\tilde{z}-(\tilde{y}\cdot \tilde{z})\tilde{y})(\tilde{y}\cdot \tilde{z})\tilde{y}{]}_{j}=\left(\sum_{k=1}^{n}{\tilde{z}}_{k}{\tilde{y}}_{k}\right){\tilde{z}}_{j}{\tilde{y}}_{j}-\left(\sum_{k,l=1}^{n}{\tilde{z}}_{k}{\tilde{z}}_{l}{\tilde{y}}_{k}{\tilde{y}}_{l}\right){\tilde{y}}_{j}^{2}\\ & =\left(\sum_{k=1,k\neq j}^{n}{\tilde{z}}_{j}{\tilde{z}}_{k}{\tilde{y}}_{j}{\tilde{y}}_{k}\right)-\left(\sum_{k,l=1,k\neq l}^{n}{\tilde{z}}_{k}{\tilde{z}}_{l}{\tilde{y}}_{k}{\tilde{y}}_{l}\right){\tilde{y}}_{j}^{2}+\left({\tilde{z}}_{j}^{2}-(\tilde{z}\cdot \tilde{y}{)}^{2}\right){\tilde{y}}_{j}^{2}, \end{array}$$

where we can use the same argument as for equation (C 5) to show that the last summand integrates to zero. Since also $\int_{S^{n-2}}{\tilde{z}}_{j}{\tilde{z}}_{k}\;dH^{n-1}(z)=0$ for any j≠k , we derive equation (C 6). Togethepossible to interpret it as a forward Euler discretizationr with equations (C 4) and (C 5), this yields

$$\begin{array}{rl} \int_{S^{n-2}}{\left[R(\theta {)}^{T}\tilde{z}\right]}_{j}^{2}\;\;dH{,}^{n-1}(z) & =\tilde{C}\left(1-y_{j}^{2}\right). \end{array}$$

The statement now follows from substituting the above into equation (C 3), with constants given by

$$\begin{array}{rl} & C_{2}=\frac{\left|S^{n-2}\right|}{\left|S^{n-1}\right|}\int_{0}^{\pi }e^{\cos\;\varphi }\cos^{2}\;\varphi \;\sin^{n-2}\;\varphi \;\;d\varphi -C_{3},\;C_{3}=\frac{\tilde{C}}{\left|S^{n-1}\right|}\int_{0}^{\pi }e^{\cos\;\varphi }\sin^{n}\;\varphi \;\;d\varphi . \end{array}$$

Since $\tilde{C}>0$ for all n⩾2 , it directly follows that $C_{3}>0$ . To show that $C_{2}>0$ for all n⩾2 , we first show that $\tilde{C}=\left|S^{n-2}|/(n-1\right)$ . For n=2 , this follows directly from $\tilde{C}=|S^{0}|=2$ . For n>2 , we have

$$\begin{array}{r} \tilde{C}=|S^{n-3}|\int_{0}^{\pi }\cos^{2}\varphi \sin^{n-3}\varphi \;\;d\varphi \;=\;|S^{n-3}|\int_{0}^{\pi }\sin^{n-3}\varphi -\sin^{n-1}\varphi \;\;d\varphi . \end{array}$$

Using integration by parts, we further derive that

$$\begin{array}{r} \int_{0}^{\pi }\sin^{n-1}\varphi \;\;d\varphi \;=\;(n-2)/(n-1)\sin^{n-3}\varphi \;\;d\varphi . \end{array}$$

As shown in lemma B.1, the recursive form of the Jacobian determinant of spherical coordinates yields that

$$\left|S^{n-2}\right|=\left|S^{n-3}\right|\int_{0}^{\pi }\sin^{n-3}\varphi \;d\varphi$$

Combining these equalities, we see that

$$\begin{array}{r} \tilde{C}=(1-((n-2)/(n-1)))|S^{n-2}|=|S^{n-2}|/(n-1), \end{array}$$

and therefore, with integration by parts, we get

$$\begin{array}{rl} C_{2} & =\frac{|S^{n-2}|}{|S^{n-1}|}\int_{0}^{\pi }e^{\cos\varphi }\left[\cos^{2}\varphi \sin^{n-2}\varphi -\frac{1}{1-n}\sin^{n}\varphi \right]\;\;d\varphi \\ & =\frac{|S^{n-2}|}{|S^{n-1}|}\int_{0}^{\pi }e^{\cos\varphi }\sin^{n-2}\varphi \cos\varphi \left(\cos\varphi -1\right)\;\;d\varphi . \end{array}$$

Due to the symmetry of sine and cosine, we get

$$\begin{array}{rl} C_{2} & =\frac{|S^{n-2}|}{|S^{n-1}|}\int_{0}^{\pi /2}e^{\cos\varphi }\sin^{n-2}\varphi \cos\varphi \left(\cos\varphi -1\right)+e^{-\cos\varphi }\sin^{n-2}\varphi \cos\varphi \left(\cos\varphi +1\right)\;\;d\varphi \\ & =2\frac{|S^{n-2}|}{|S^{n-1}|}\int_{0}^{\pi /2}\sin^{n-2}\varphi \left(\cos(\varphi )\cosh(\cos(\varphi ))-\sinh(\cos(\varphi ))\right)>0, \end{array}$$

where the positivity follows from the fact that the function t↦tcosh⁡(t)−sinh⁡(t) is positive for t>0 and both sine and cosine are positive for φ∈(0,π/2) .∎
